# High Polarity Doping of CoFe Layered Hydroxides: Bifunctional and Corrosion-Resistant Anion Exchange Membrane Seawater Electrolyzers

**DOI:** 10.1007/s40820-026-02230-8

**Published:** 2026-06-01

**Authors:** Anandhan Ayyappan Saj, Sampath Prabhakaran, Mohsin Rasool, Kousik Bhunia, Dongho Lee, Hyunseok Ko, Tukaram D. Dongale, Muthukumar Perumalsamy, Arul Saravanan Raaju Sundhar, Do Hwan Kim, Sang Jae Kim

**Affiliations:** 1 Nanomaterials & System Lab, Major of Mechatronics Engineering, Faculty of Applied Energy System, Jeju National University, Jeju, 63243 South Korea; 2 Nanomaterials & System Lab, Major of Mechanical System Engineering, College of Engineering, Jeju National University, Jeju, 63243 South Korea; 3 Green Hydrogen Glocal Leading Research Center (gH2-RC), Jeju National University, Jeju, 63243 Republic of Korea; 4 Research Institute of New Energy Industry (RINEI), Jeju National University, Jeju, 63243 South Korea; 5 Division of AI Convergence Research, Korea Institute of Ceramic Engineering and Technology (KICET), Jinju, 52851 Republic of Korea; 6 University of Ulsan, Ulsan, 44776 Republic of Korea; 7 Division of Science Education and Institute of Fusion Science, Department of Energy Storage/Conversion Engineering (BK21 FOUR), Jeonbuk National University, Jeonju, Jeonbuk 54896 Republic of Korea; 8 Computational Electronics and Nanoscience Research Laboratory, School of Nanoscience and Biotechnology, Shivaji University, Kolhapur, Maharashtra 416004 India

**Keywords:** Corrosion resistance, F-doping, H_2_ generation, Seawater electrolysis, Long short-term memory (LSTM)

## Abstract

Green hydrogen production through seawater electrolysis is a promising strategy, although challenges such as sluggish oxygen evolution reaction (OER) kinetics and chlorine (Cl^−^) corrosion hinder its practical applicability. A novel fluorine (F)-doped cobalt (Co) and iron (Fe) layered metal hydroxide (F-CoFe LMH-8) is developed as a robust bifunctional catalyst achieving 81.23 and 265.5 mV at 10 mA cm^−2^ for hydrogen evolution reaction (HER) and oxygen evolution reaction (OER), respectively. Theoretical and experimental studies demonstrate that the F-doping modulates the electronic structure, effectively tuning Fe sites toward a high-spin configuration that optimizes binding energies and induces a chlorophobic effect that repel corrosive (Cl^−^) ions. Notably, the F-CoFe LMH-8( +|| −) bifunctional catalyst integrated anion exchange membrane water electrolyzer (AEMWE) exhibited outstanding performance for continuous H_2_ production, achieves a current density of 1.2 A cm^−2^ in 1 M KOH, 1.02 A cm^−2^ in 1 M KOH + 0.5 M NaCl, and 1 A cm^−2^ in 1 M KOH in seawater at 2.3 V. Furthermore, a long short-term memory-based machine learning model was employed to forecast and predict the stability of F-CoFe LMH-8. This approach provides a comprehensive pathway for heuristic design of durable, chlorophobic, and advanced electrocatalyst for seawater-based AEMWE and large-scale hydrogen production.

**Supplementary Information:**

The online version contains supplementary material available at 10.1007/s40820-026-02230-8.

## Highlights

- CoFe layered metal hydroxide (LMH) was synthesized via a MgO nanoparticle assisted method, fluorine (F) doping effectively modulated the electronic structure of CoFe LMH by selectively tuning Fe sites while preserving Co integrity, resulting in a high spin Fe configuration conducive to enhanced catalytic activity.
- F-CoFe LMH-8 as a bifunctional catalyst , exhibiting strong hydrophilic and oxophilic behavior while effectively repelling chloride ions (chlorophobic) in seawater conditions.
- F-CoFe LMH-8 (+||–) anion exchange membrane device delivered a current density of 1 A cm^–2^ at 2.3 V in seawater and maintained exceptional operational durability, exhibiting a degradation rate of only 0.15 μV h^–1^ over 500 h of continuous operation.

**Supplementary Information:**

The online version contains supplementary material available at 10.1007/s40820-026-02230-8.

## Graphical abstract

**Figure:**
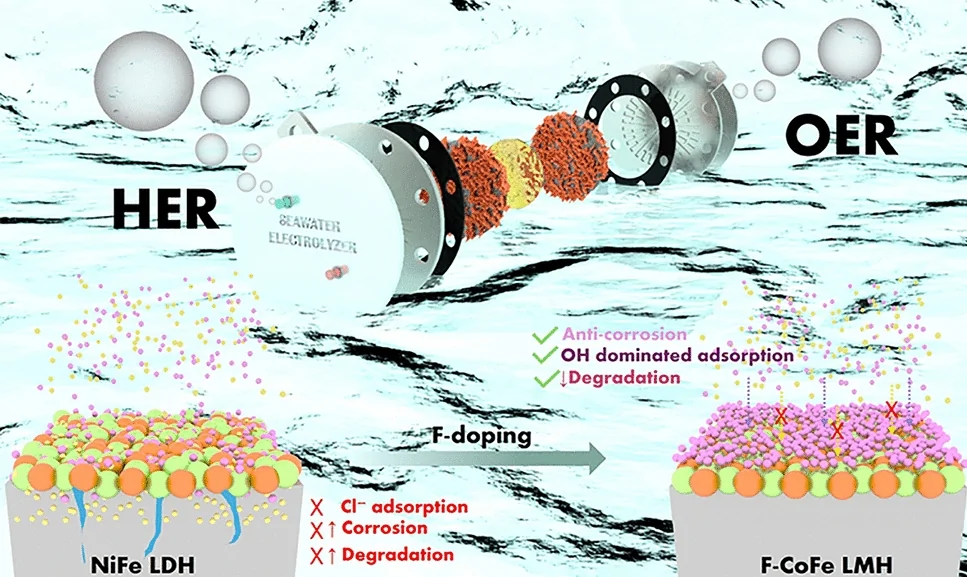

## Introduction

The excessive consumption of fuels and the detrimental environmental problems that emerged during the past human endeavors compel us to explore clean and sustainable energy resources for future energy demands [1–4]. Hydrogen economy through water electrolysis is a future-proof technology since the generation and combustion product for hydrogen is simply water [5]. In commercial water electrolyzers, noble metals like Pt and Ir/Ru are used to facilitate the hydrogen/oxygen evolution half-reactions (HER/OER) [6, 7]. Water electrolysis is a promising technology, yet scaling up is heavily hindered by its cost, stability, activity, and excessive amount of freshwater demand [8]. To eliminate the cost and freshwater demand, direct electrolysis of seawater has emerged as an illimitable raw material for green hydrogen production [9–11]. Despite its unlimited advantages of seawater electrolysis, anode catalysts are hindered due to competing complex chloride evolution reaction (CIER) and sluggish OER. Therefore, a highly efficient and OER-selective anodic catalyst is the ultimate requirement for seawater electrolysis [12]. The strong interaction between Cl^−^ ions and catalyst active site accelerates catalyst corrosion in the saline environments. Moreover, the operational voltage employed in commercial water electrolyzer systems at current densities (0.1–1 A cm^−2^) adds extra unwanted stress at the electrocatalytic interface [13].

In recent year, alternative strategies have been explored that can achieve high activity, excellent stability, and lower cost for an electrolyzer. Transition metal hydroxide-based catalyst, due to their abundance and electrocatalytic activity, is a promising avenue which are being explored as a potential alternative [1, 5, 14, 15]. Currently, various nitrides [16, 17], oxides [18, 19], phosphides [20], and sulfides [21] have been reported for seawater electrolysis. These anodes normally undergo phase-transformative reconstructions, during which create surface defects that provide opportunities for chlorine (Cl^−^) attacks [22, 23]. Therefore, these anodes become challenging to operate stably, because they experience gradual degradation and their disordered anion distribution can reduce OER activity by affecting intermediate adsorption.

Major focus has been placed on regulating the doping and intercalation of anions into layered double hydroxide (LDH)-based anodes in alkaline seawater electrolyzers. Electrode protection by introducing anions at the anode interface has been proposed, and its feasibility has been demonstrated in the published literatures, where LDH-based materials were intercalated with various anions [22]. For example, Cl^−^ ions are introduced in the NiFe sites, although the catalyst only performed for 100 h of stable operation @ 200 mA cm^−2^ current density @ 30% KOH seawater in 80 °C [24]. Regulating electronic structure and local coordination environment of Fe sites seems to play a key role in optimizing catalytic performance in seawater. Fluorine incorporation into NiFe LDH has been previously employed to stabilize high-valent Fe sites; however, the Pt‖F–NiFe AEM device exhibited relatively low operating voltage of 2.04 V at 500 mA cm^−2^ and maintained stability for only 80 h in seawater. A sulfate (SO_4_^2−^) coating was applied through surface reconstruction from la-S-NiFe-LDH/NFF where device exhibited operational stability for only 120 h [25]. Sulfate (SO_4_^2−^) ions were also intercalated through a buffer solution in CoFe LDH, although the Pt || CoFe LDH/(SO_4_^2−^) device exhibited high overpotentials of 2.51 V at 1.0 cm^−2^ at 60 °C indicating inadequate catalytic performance [26]. Anodes have also been reported to be intercalated with aromatic small molecules to boost its operational time; although this approach extended the operational time of the alkaline seawater electrolyzer to 220 h, the cell exhibited suboptimal operational performance, requiring voltages above 2.5 V at 65 °C to reach 1.0 A cm^−2^ [27].

In this work, we developed a simple, scalable, and cost-effective synthetic route for producing metal hydroxide nanostructures, which can be readily expanded to large-scale reactions. F was employed as the charge-balancing ion functioning as an effective weak field ligand, serving as doping/intercalant species that selectively occupies Fe-centered coordination sites while leaving the Co-based active centers intact. This targeted modulation Fe electronic environment, stabilizes high-spin Fe–O states, expands the lattice framework, and enables sustained OH^–^ accessibility at the catalytic interface. The resulting electronic configuration also provides strong resistance against chloride-induced degradation, thereby enhancing the catalyst’s operational durability under alkaline seawater electrolysis conditions. Overall, this synthesis platform offers a practical pathway toward large-scale manufacturing of high-performance electrocatalysts suitable for industrial alkaline and seawater electrolysis applications.

## Experimental Section

### Synthesis of CoFe LMH and F-CoFe LMH

At first, cobalt nitrate hexahydrate and iron sulfate hexahydrate were dissolved one-by-one in 25 mL of deionized (DI) water and stirred at room temperature for 10 min. Then, MgO nanoparticle (NPs) powder was added to the solution slowly and stirred at room temperature for 1 h, after which the catalyst was kept uninterrupted for 24 h. The reaction mixture was centrifuged at 5000 rpm for 3 min, and the supernatant was discarded. The solid product obtained after centrifugation was washed repeatedly with DI water and ethanol until a pure powder was obtained. Finally, the synthesized powder is dried in hot-air oven at 60 °C overnight.

For F-CoFe LMH synthesis, 25 mL of 1 M (ammonium fluoride) NH_4_F in DI water is used instead of pure DI water, while keeping the other synthesis parameters intact. The precursor concentrations taken for the synthesis of the catalyst and their corresponding catalyst names are outlined in Table S1.

## Results and Discussion

### Structural Characterizations

**Fig. 1:**
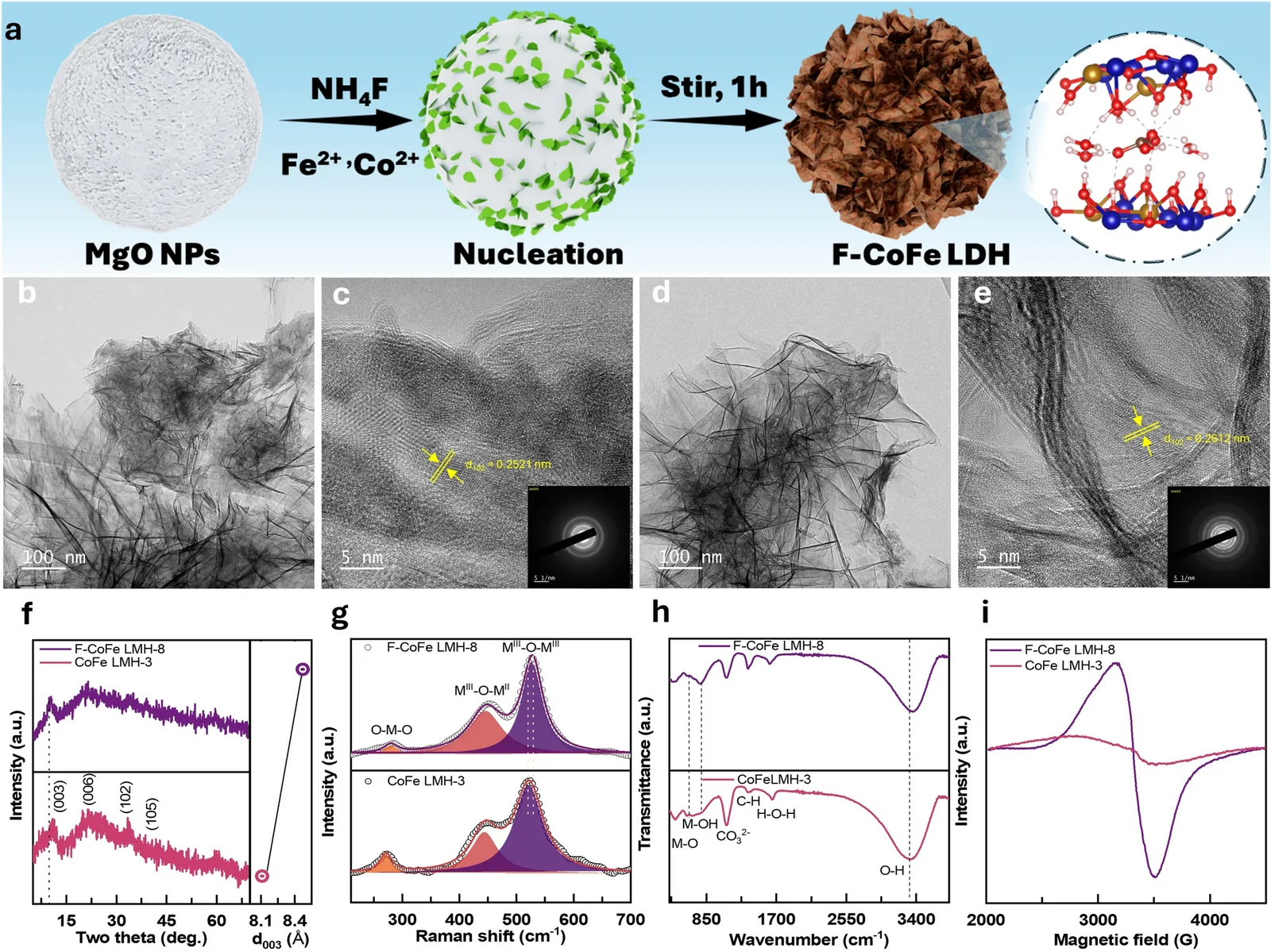
**a** Schematic representation of synthesis. HR-TEM images at various magnifications of **b, c** CoFe LMH-3 and **d, e** F-CoFe LMH-8 and their corresponding SAED patterns (inset). **f** P-XRD analysis and corresponding d-spacing, **g** Raman spectra, **h** FT-IR spectra, and **i** EPR analysis for CoFe LMH-3 and F-CoFe LMH-8

F-doped CoFe LMH nanostructured sheets were fabricated using a one-step ion exchange method using MgO NPs as a template, as illustrated in Fig. 1a. Initially, MgO NPs react with water to form surface hydroxyl species, which gradually transform into magnesium hydroxide (MgOH_2_). These Mg(OH)_2_ layers act as a sacrificial template, enabling ion exchange with cobalt and iron ions in solution. During this process, Co^2+^/Co^3+^ and Fe^2+^/Fe^3+^ incorporate into the hydroxide framework, forming layered CoFe hydroxides, while Mg^2+^ ions are released into the solution. To monitor the structural evolution of the catalyst, aliquots were periodically retrieved throughout the synthesis and analyzed via scanning electron microscopy (SEM) and powder X-ray diffraction (XRD) (Figs. S1 and S2). SEM imaging revealed the progressive formation of a sheet-like morphology. Concurrently, XRD profiles indicated significant reconstruction, evidenced by the emergence of new diffraction peaks corresponding to newly formed crystallographic planes. A series of fluorine-doped and undoped catalysts with various stoichiometric ratios was synthesized. The SEM analysis of these catalysts revealed that they exhibited a nanosheet-like morphology, highlighting their distinct structural features. The energy-dispersive analysis X-ray (EDAX) and elemental mapping were performed to confirm the uniform distributions of the constituting elements in the synthesized catalyst (Figs. S3–S12). TEM analysis of CoFe LMH-3 showed a nanosheet-like morphology, found to be identical with the SEM analysis (Fig. 1b). The d-spacing of the CoFe LMH-3 is found to be 0.2521 nm which is consistent with the (102) planes of CoFe LMH-3 (Fig. 1c). Similarly, TEM images of the F-CoFe LMH-8 are well matched with the SEM images (Fig. 1d). After F-doping, the d-spacing is increased to 0.2612 nm for the F-CoFe LMH-8 (Fig. 1e). Additionally, ring pattern of selected area electron diffraction (SAED) reveals the polycrystalline nature of the doped and undoped catalyst (inset of Fig. 1c, e respectively). The elemental mapping of CoFe LMH-3 and F-CoFe LMH-8 demonstrated uniform distribution of Co, Fe, O, and F, respectively (Figs. S13 and S14).

The crystallinity of the catalyst was characterized by powder X-ray diffractometer (XRD). The powder XRD of the synthesized catalyst is presented in Figs. 1f and S15. The XRD pattern exhibited well-defined diffraction peaks position at 10.47°, 21.69°, 33.7°, and 39.0° is corresponding to the (003), (006), (102), and (105) plane of the CoFe LMH [ICSD file No. 00-050-0235 for CoFe(OH)_2_; 00-046-0605 for Co(OH)_2_; and ICSD pattern of 98-011-0293 for Fe(OH)_2_]. With varying the Co and Fe ratio in addition with the F-doping, diffraction peaks gradually shifted to the lower two theta value. The downshift in 2θ values can be attributed to lattice expansion induced by high-spin configurations. Moreover, a peak shift was identified on the modified catalyst, which can be attributed to F-doping, verifying the fact that F was only doped into the lattice without forming other separate crystallite phases [28–30]. The XRD patterns downshift shows d-spacing value obtained from (003) plane of F-CoFe LMH-8 (8.45 Å) as compared to CoFe LMH-3(8.10 Å) to be increasing suggesting that anionic molecules occupy the interlayer spaces relative to the case of alpha species which corresponds to the observation made through HR-TEM. The doped and undoped catalyst were further investigated by Raman spectroscopy as presented in Figs. 1g and S16. The CoFe LMH-1 exhibited three distinct vibrational peak position at 250, 437.2, and 507.8 cm^−1^. The vibration band position at 250 cm^−1^ originates from the O–M–O (M-Metal) bond bending (E_g_) or rocking mode, whereas the Raman shift position at 437.2 cm^−1^ originates from the O^III^–M–O^II^ bond asymmetric stretching (E_g_) vibration. The most intense vibration band appeared at 507.8 cm^−1^ corresponding to the O^III^-M-O^III^ bond symmetric stretching (A_1__g_) mode of vibration. However, the vibration band is found to be shifted higher value with increasing the Fe content for the CoFe LMH-1 to CoFe LMH-3. However, further increasing the Fe concentration, vibration band intensity corresponding to the 437.2 and 507.8 cm^−1^ is significantly decreased and or disappeared, whereas new vibrational bands appeared at 270 and 635.6 cm^−1^. The vibrational band 207.1 cm^−1^ attributed to Fe–OH bending vibrations and 635 cm^−1^ arising from M–O symmetric stretching M–O bond for the CoFe LMH-4. The vibration band shift further observed for the CoFe LMH-5 with increasing Fe content. The Raman analysis indicated that at low Fe concentration, Co–OH/Co–O bond vibration is dominated, as observed from the from the peak shift. However, at higher Fe concentration in the CoFe LMH catalyst, the Fe–OH/Fe–O bond vibration is dominated [31, 32]. Moreover, similar trend is also found after the F-doping, indicating that the core band structure of the synthesized catalyst remains unaffected by F-doping. The apparent shift after F-doping indicates that the F-doping tune the M–O/M–OH bond strength.

FT-IR spectroscopy was employed to analyze the surface properties of the doped and undoped sample as presented in Figs. 1h and S17. The changes could be detected among the catalysts, where the absorption bands at 3317, 1658, 1366, 1106, 657, and 473 cm^−1^ could be assigned to the vibration bands corresponding to the OH, H–O–H, C–H, CO_3_^2−^, M-OH, and M–O, respectively. The absorption peak from 2800 to 3680 cm^−1^ is the distinguishing peak of bulk hydroxyl and surface hydroxyl representative of the oxygen stretching vibration of hydroxyl and water molecules in the interlayer, including hydrogen-bonded water. The characteristic absorption peak present at 1366 cm^−1^ and 1106 cm^–1^ can be attributed to presence of C–H and carbonate (CO_3_^2−^) in the interlayer of the catalyst. Furthermore, the characteristic absorption between 1000 and 500 cm^−1^ can be attributed to the metal oxygen (M–O) and metal hydroxyl stretching (M-OH). To investigate whether doping-induced defects and d-orbital electronic structure modulation, electron paramagnetic resonance (EPR) spectroscopy was employed (Fig. 1i). The EPR signals reveals that the CoFe LMH-8 exhibited a higher peak intensity as compared to CoFe LMH-3, which indicated a higher number of unpaired electrons present in the F-doped catalyst. The high peak intensity of the F-CoFe LMH-8 signifies a high-spin configuration, which is expected to be more beneficial for enhanced electrocatalytic activity [33–35].

**Fig. 2:**
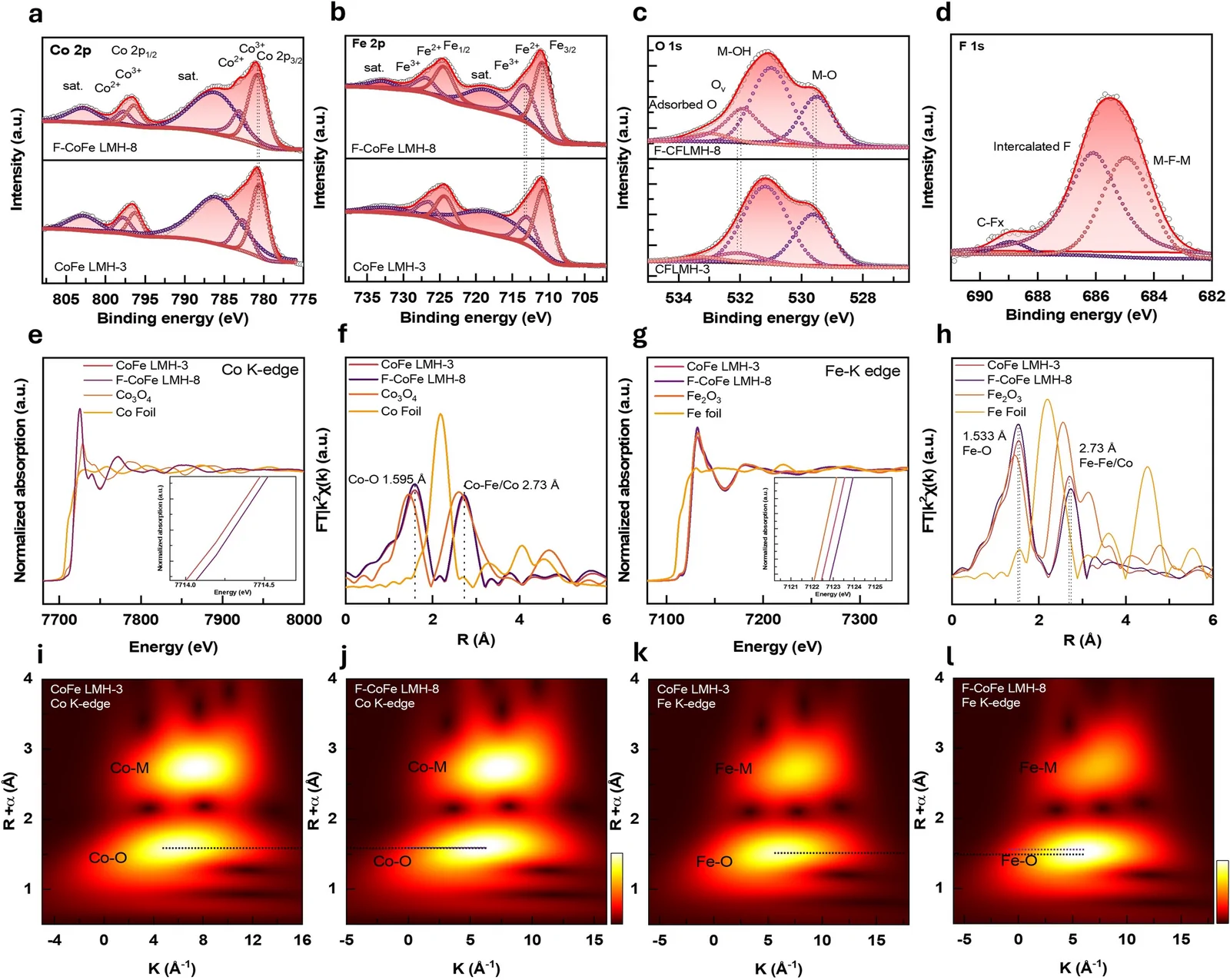
XPS core level spectra of **a** Co, **b** Fe, **c** O, and **d** F of CoFe LMH-3 and F-CoFe LMH-8. The XANES spectrum of **e** Co K-edge and **f** corresponding EXAFS. XANES spectrum of **g** Fe K-edge and **h** corresponding EXAFS. WT-EXAFS of Co K-edge **i** CoFe LMH-3, **j** F-CoFe LMH-8 and Fe K-edge, **k** CoFe LMH-3 and** l** F-CoFe LMH-8

X-ray photoelectron spectroscopy (XPS) was employed to characterize the chemical oxidation/compositional state disparities between CoFe LMH-3 and F-CoFe LMH-8. The survey spectrum of the catalyst is presented in Fig. S18. The high-resolution XPS of Co 2*p* is presented in Fig. 2a. The Co 2*p* XPS is deconvoluted to two distinct binding energy (BE) peaks, displaying the co-existence of Co (III) (780.58 eV (Co 2*p*_3/2_) and 796.30 eV (Co *2*p_1/2_)) and Co (II) at (782.70 eV (Co 2*p*_3/2_) and 797.89 eV (Co 2*p*_1/2_)) state. The pair of peaks at 786.35 and 802.84 eV is ascribed to the multi-electron shake up satellite peaks. However, the BE is shifted around 0.24 eV for the F-CoFe LMH-8 catalyst compared to CoFe LMH-3 after F-doping, suggesting that the incorporation of F slightly alters the electronic structure of cobalt [36]. The Fe 2*p* XPS shows BE peaks at 713.06 and 726.63 eV corresponding to the Fe (III) oxidation state and BE peaks at 710.61 and 724.39 eV corresponding to the Fe (II) oxidation state of Fe. Moreover, BE peaks at 718.91 and 733.83 eV are ascribed to the multi-electron shake up satellite peaks (Fig. 2b) [37]. The BE value of Fe 2*p* is shifted to 0.45 eV for F-CoFe LMH-8 compared to the CoFe LMH-3 suggesting that F-doping significantly tunes the electronic environment of Fe atom. The O 1*s* XPS is deconvoluted to four distinct BE peaks (Fig. 2c). Specifically, the BE peak positioned at 529.58, 531.18, 532.08, and 533.18 eV is ascribed to the metal–oxygen bond (M–O bond), oxygen in hydroxyl groups (M–OH), oxygen vacancy (O_v_), and adsorbed oxygen [38, 39]. The O 1*s* BE value is shifted 0.12 eV to higher energy after F-doping, indicating electron migration from O to F due to the higher electronegativity of F. The F 1*s* signature of the F-CoFe LMH-8 catalyst indicates successful incorporation of F into the CoFe LMH catalyst. The high-resolution F 1*s* XPS is presented in Fig. 2d. The deconvoluted F 1*s* spectrum revealed two distinct BE region. The BE value ~ 684.5 eV is attributed to metal–fluoride (M–F) bonds, indicating substitutional F-doping into the Co/Fe layered hydroxide lattice. Moreover, BE peak positions at ~ 688.5 eV, corresponding to carbon–fluorine (C–F) bonds, likely arising from surface-adsorbed or intercalated fluorine-containing species [40]. Furthermore, to ascertain the role of F in F–CoFe LMH-8, its chemical signature was analyzed through ^19^F MAS NMR (Fig. S19). The spectrum revealed a broad response, indicative of a distribution of absorbed and substituted sites (F atoms for OH^−^ ions). This observation is consistent with F 1*s* XPS and XRD analyses, confirming the presence of fluorine both as a dopant and as an intercalant [41, 42].

X-ray absorption near edge structure (XANES) and extended X-ray absorption fine structure (EXAFS) were conducted to investigate the electronic structure and coordination environment of Co and Fe of CoFe LMH-3 and F-CoFe LMH-8. Barely minimal changes in the XANES spectra of Co K-edge energy are found after F-doping (Fig. 2e). The coordination environment of Co-site is believed to be intact. The bond lengths of Co–O (1.595 Å) and Co–Co/Fe (2.73 Å) estimated from the EXAFS spectra are found to be similar for both the CoFe LMH-3 and F-CoFe LMH-8 catalyst (Fig. 2f) [4]. However, XANES of Fe K-edge spectra significantly is shifted to higher energy after F-doping. The upshift of Fe K-edge spectra suggested the formation of higher valent Fe metal centers (Fig. 2g). The Fe K-edge EXAFS analysis revealed a pronounced alteration in the local coordination environment upon doping. A shift was observed after doping and the presence of Fe–O (1.533 Å) is found to be more dominant and Fe–Fe/Co (2.73 Å) bond is suppressed (Fig. 2h). The Fourier-transform k^3^ weighted wavelet-transformed (WT)-EXAFS of Co shows two Co–O and Co–Co/Fe coordination peaks located at 1.5 and 2.7 Å. The Co–O and Co-M coordination peaks remain essentially unchanged after F-doping, indicating that the Co active sites are structurally preserved and largely unaffected by F incorporation (Fig. 2i, j). Furthermore, the WT-EXAFS analysis of Fe reveals an increased intensity for the Fe–O coordination at ~ 1.5 Å and a corresponding decrease in the Fe–Fe/Co scattering contribution at ~ 2.7 Å along with a shift toward higher R-space of Fe–O after F-doping (Fig. 2k, l). This indicates a strengthened Fe–O local environment and a partial disruption of the extended metal–metal network. Magnetic hysteresis (M-H) analysis revealed that F-CoFe LMH-8 possesses an enhanced saturation magnetization relative to CoFe LMH-3. Additionally, F-CoFe LMH-8 exhibited an effective magnetic moment of 4.125 μB, confirming the formation of a high-spin state configuration (Fig. S20). As fluorine functions as a weak-field ligand, its incorporation suppresses medium-spin electron pairing configurations and stabilizes a high-spin configuration. The M–O high-spin bond environment regulation of the F-CoFe LMH-8 strengthens the M *3d*-O *2p* hybridization through spin-conserved electron transfer, which enhances HER/OER-kinetics and selectivity [22, 43].

### Electrocatalytic Performance for HER

**Fig. 3:**
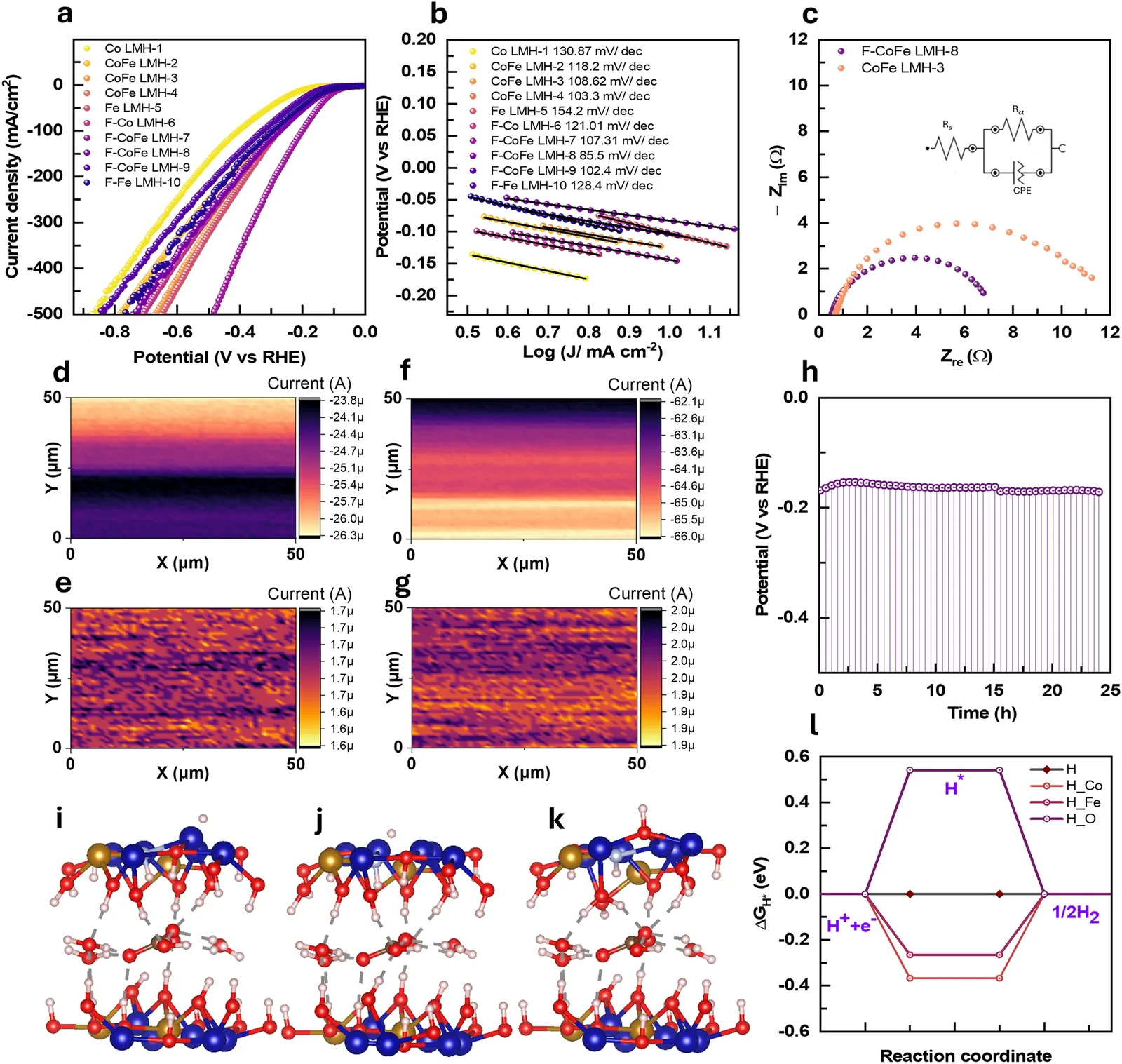
**a** LSV profile and **b** Tafel slope of the different composition F-doped and undoped FeCo LMH catalyst. **c** Nyquist plot of CoFe LMH-3 and F-CoFe LMH-8. SECM analysis of CoFe LMH-3 catalyst **d** surface and **e** tip current profile. SECM analysis of F-CoFe LMH-8 catalyst **f** surface and **g** tip current profile. **h** Electrochemical stability of F-CoFe LMH-8 at − 50 mA cm^−2^ constant current density. H* adsorption energy at **i** Co-site, **j** Fe-site, and **k** O-site of F-doped FeCo LMH catalyst. **l** H* adsorption Gibbs free energy on Co, Fe, and O-sites

To verify the effect of electronic structure modulation toward enhanced electrocatalytic activity, the HER performance was evaluated in 1 M KOH using a standard three-electrode system. Before measuring linear sweep voltammetry (LSV) and electrochemical impedance spectroscopy (EIS), a cyclic voltammetry (CV) at 50 mV s^−1^ scan rate was performed to stabilize the electrode. The LSV profile of the synthesized catalyst is presented in Fig. 3a. The F-CoFe LMH-8 catalyst exhibited tremendous activity, required overpotential of only 81.23 mV @10 mA cm^−2^ among the as-synthesized catalyst, far superior to the to the other undoped and doped catalyst such as CoFe LMH-3 (122.73 mV), CoFe LMH-1 (196.89 mV), CoFe LMH-2 (132.5 mV), CoFe LMH-4 (110.83 mV), CoFe LMH-5 (133.11 mV), F-CoFe LMH-6 (144.7 mV), F-CoFe LMH-7 (118.76 mV), F-CoFe LMH-9 (105.65 mV), and F-CoFe LMH-10 (115.41 mV). The relative catalytic performance comparison is presented in Fig. S21. The Tafel slope of the catalyst is estimated from LSV profile to analyze the reaction kinetics (Fig. 3b). The lower Tafel slope value of F-CoFe LMH-8 (85.5 mV dec^−1^) is associated with the faster kinetics compared to CoFe LMH-3 (104.4 mv dec^−1^). The EIS was analyzed to determine the charge transfer kinetics of the catalyst by applying − 50 mV. The Nyquist plots along with equivalent circuit diagram are presented in Fig. 3c. The solution resistance (R_s_) and charge transfer resistance (R_ct_) are found to be lower for the F-CoFe LMH-8 (R_s_ = 0.487 Ω and R_ct_ = 6.77 Ω) compared to the CoFe LMH-3 (R_s_ = 0.604 Ω and R_ct_ = 11.069 Ω) which is well aligned with the LSV signatures. To analyze micro-kinetics of the catalyst, in situ scanning electrochemical microscopy (SECM) was employed. The surface (I_s_) and tip (I_t_) currents at the applied potential around the HER region are presented in Fig. S22. The SECM measurements of CoFe LMH-3 were performed at applied potential of − 1.1 V vs Ag/AgCl; the substrate micro-currents response is found to be in the range of I_s_ of − 23.8 – − 26.3 μA along with the I_t_ in found to be 1.6 - 1.7 μA. The I_s_/I_t_ increases gradually with increasing the applied potential (Figs. 3d, e and S23), whereas F-CoFe LMH-8 showed a micro-current response of I_s_ of − 66 – − 62.1 μA and the corresponding I_t_ of 1.9 – 2.0 μA (Figs. 3f, g and S24). The higher I_s_/I_t_ response of the F-CoFe LMH-8 indicates a significant electronic structure regulation after F-doping, resulting the remarkable activity of F-CoFe LMH-8. A performance comparison table compiling previously reported non-precious metal-based HER catalyst in alkaline electrolyte is presented in Table S2.

Long-term catalytic activity is analyzed by chronopotentiometry (V-t plots) measurements by applying a constant current density of − 50 mA cm^−2^ for 24 h. The voltage response at the applied current density remained constant throughout the stability test without any degradation, indicating excellent durability of the as-synthesized F-CoFe LMH-8 catalyst (Fig. 3h). The LSV profile measured after the stability also shows negligible degradation of the LSV performance (Fig. S25). To analyze the physicochemical changes after the electrochemical stability test, the FT-IR and Raman spectroscopy were performed (Figs. S26 and S27). The FT-IR shows a characteristic shift in the OH vibration band, due to the removal of water molecules present in the interlayer of M-OH, and the upshift representative of the free, non-H bonded hydroxyl ions. The Raman spectra reveals that the vibration band 587 cm^−1^ corresponding to the M^II^ –O_A1g_ bond vibration broadens and shifts to higher wavenumber, signifying the formation of higher valent oxygenated surface species (oxy-hydroxide and or oxide) formed during the HER stability test.

Spin-polarized density-functional theory (DFT) calculations were performed to gain atomic insights into the possible origin of the preeminent performances of F-CoFe LMH-8 toward HER. A F@CoFe LMH-8 model slab was constructed to quantify the effect of an electronegative F perturbs site-specific HER activity. The computational modeling settings and convergence criteria are described in the supporting information. The Gibbs free energy of H* intermediate adsorption (ΔG_H*_) serves as a decisive descriptor for determining of the HER activity. The free energies referenced to RHE at 298.15 K within the computational hydrogen electrode. The H* adsorption models on the three different adsorption sites are presented in Figs. 3i–k and S28. For the HER, the redistribution manifests in distinct adsorption thermodynamics: ΔG_H*_ = − 0.367 eV for Co, − 0.265 eV for Fe, and 0.541 eV for O (Fig. 3l). The near-thermoneutral value, the Fe-site identifies as the kinetically preferred H* site, whereas Co-site and O-site is found to be over-binds and under-binds, respectively, both deviating from the ideal descriptor window. The role of F is twofold: It withdraws charge from Fe and Co, enhancing metal–F/O polarizations, while simultaneously tuning the Fe-site into the HER active center.

### Electrocatalytic Performance for OER

**Fig. 4:**
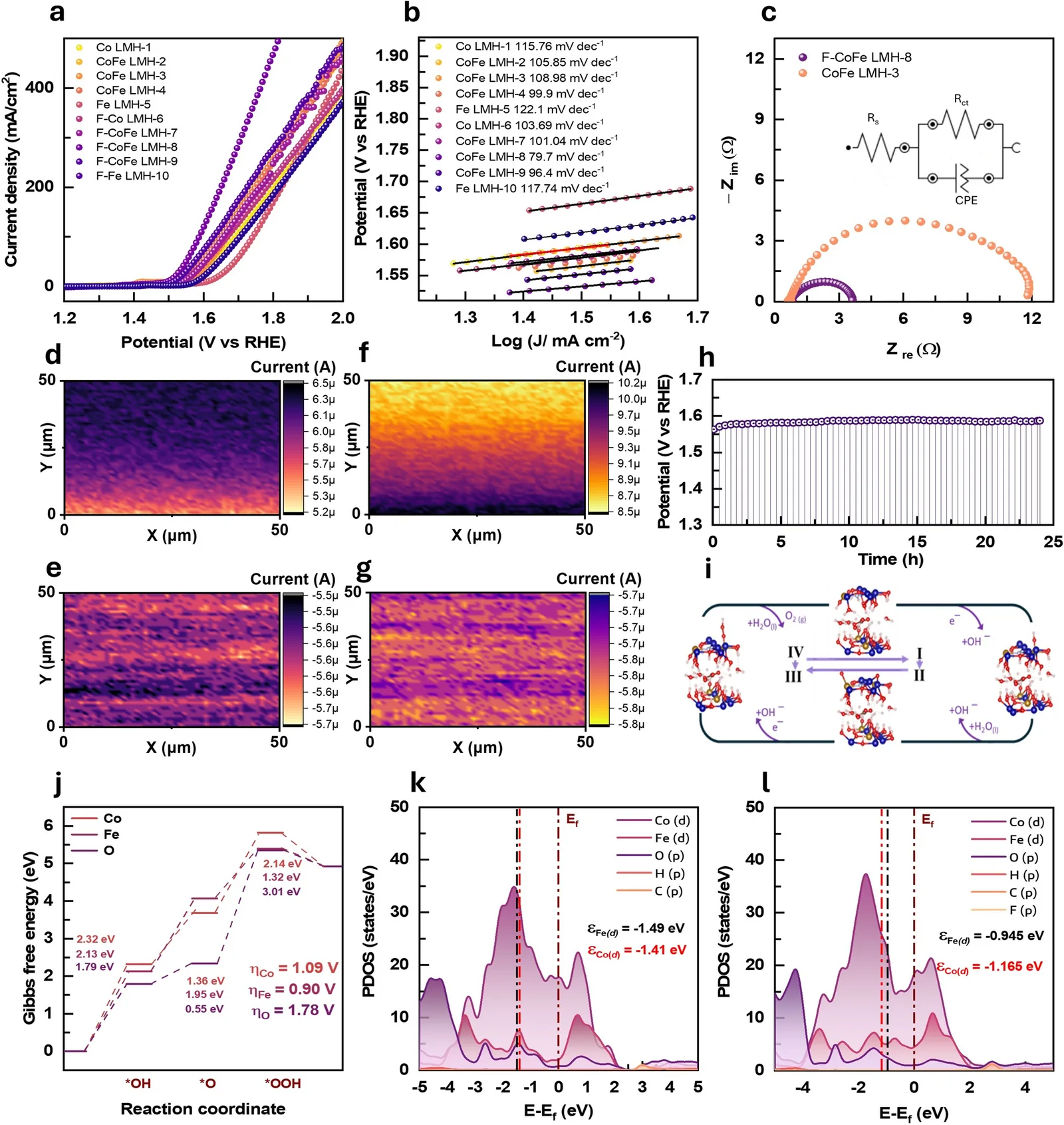
**a** LSV analysis, **b** Tafel slope for CLMH, CoFe LMH-1 CoFe LMH-2, CoFe LMH-3, CoFe LMH-4, FLMH-5, F-CLMH-6, F-CoFe LMH-6, F-CoFe LMH-7, F-CoFe LMH-8, F-CoFe LMH-9, and F-FLMH-10. **c** Nyquist plot for CoFe LMH-3 and F-CoFe LMH-8. SECM analysis for CoFe LMH-3 **d** surface, **e** tip. SECM analysis for CoFe LMH-3 for F-CoFe LMH-8 **f** surface, **g** tip. **h** Stability for F-CoFe LMH-8, **i** OER reaction mechanism. **j** Gibbs free energy diagram for OER for F-CoFe LMH to find the reaction coordination site. PDOS for different coordination sites for **k** CoFe LMH-3 and **l** F-CoFe LMH-8

Due to the typical four-electron transfer process, OER shows sluggish kinetics, which is regarded as one of the bottlenecks for the overall water electrolysis process. The LSV profile of the OER is presented in Fig. 4a. The order of OER performed in terms of overpotential is as follows: F-CoFe LMH-8 (265.5 mV) < F-CoFe LMH-9 (280 mV) < F-CoFe LMH-4 (304 mV) < F-CoFe LMH-7 (308 mV) < CoFe LMH-6 (310 mV) < CoFe LMH-2 (312 mV) < CoFe LMH-3 (317.1 mV) < CoFe LMH-1 (320 mV) < CoFe LMH-10 (340 mV) CoFe LMH-5 (380 mV) (Fig. S29). Tafel slope value observed for 79.7 mV/dec for F-CoFe LMH-8 is the lowest among the as-synthesized catalysts, and much lower than the undoped CoFe LMH-3 (101.9 mV dec^−1^) as presented in Fig. 4b. Moreover, enhanced activity of the catalyst can be rationalized from the electrochemical active surface area (ECSA). The electrochemical double-layer capacitance (C_dl_) of the catalytic materials was obtained by conducting CV at different scan rates (Fig. S30). The F-CoFe LMH-8 (8.17 mF cm^−2^) showed higher C_dl_ compared to the CoFe LMH-3 (5.37 mF cm^−2^). The ECSA of the catalyst is directly correlated with the number of active sites of the catalyst. In general, higher ECSA is directly correlated with higher number of exposed active sites of the catalyst. The ECSA of the F-CoFe LMH-8 (204.25 cm^2^) is found to be much higher than the CoFe LMH-3 (134.25 cm^2^), indicating that the F-doping exposed the higher number of active sites (Fig. S31a). Notably, F-doping enhanced both active site exposure and intrinsic OER activity of the catalyst, with F-CoFe LMH-8 exhibiting a superior ECSA-normalized TOF at 300 mV (2.883 × 10^−5^ s^−1^ cm^−2^) compared to the pristine CoFe LMH-3 1.098 × 10^−5^ s^−1^ cm^−2^ (Fig. S31c). The Nyquist plots of F-CoFe LMH-8 and CoFe LMH-3 were measured by applying 1.5 V vs RHE to determine the R_s_ and R_ct_ at the electrode/electrolyte interface. The CoFe LMH-3 (R_s_ = 0.6 Ω and R_ct_ = 11.74 Ω) exhibited larger semicircle than the CoFe LMH-8 (R_s_ = 0.73 Ω and R_ct_ = 3.0 Ω), indicating that the F-doping tunes the surface electronic structure of the FeCo LMH, and consequently, the reduced charge transfer resistance enhances the OER performance kinetics in Fig. 4c. Furthermore, the micro-kinetics of the catalyst analyzed by SECM at various potentials around the OER region is presented in Fig. S32. The measured surface current maps (I_s_) and tip (I_t_) showed that at 0.35 V vs. Ag/AgCl applied potential, F-CoFe LMH-8 exhibited a higher substrate and tip current response in the range of 10.3 to 8.5 μA (I_s_) and − 5.7 μA – − 5.8 μA (I_t_), respectively, whereas CoFe LMH-3 catalyst exhibited lower I_s_ (6.5 – 5.2 μA) and I_t_ (− 5.5 – − 5.7 μA), indicating the superior activity of CoFe LMH-8 (Figs. 4d–g, S33, and S34). The catalyst’s performance was also benchmarked against reported OER catalysts, as shown in Table S3. Furthermore, chronopotentiometry was performed at a fixed current density of 50 mA cm^−2^ for 24 h to investigate the durability of the synthesized catalyst. The F-CoFe LMH-8 catalyst exhibited stable voltage output over the 24 h measurement time with no degradation, demonstrating excellent stability of the catalyst (Fig. 4h). Moreover, after the stability test, LSV was performed (Fig. S35). Almost negligible changes are observed in the LSV profile before and after the stability test. Post-analysis of the catalysts was performed after the stability test to cross check phase transition after the stability test. The FT-IR and Raman analysis of the fresh and stability test performed electrode is presented in Figs. S36 and S37. The FT-IR shows a shift in the OH stretching peak, repressed due to the removal of water molecules in the inter-space, and the upshift representative of the free or non-H bonded hydroxyl ions. In Raman spectroscopy, the M^II^ –O_A1g_ broadens and upshifts, signifying the formation of more prevalent M^III^ –O_A1g_ bonds, which act as the active centers.

To identify the active site of the catalyst for OER, theoretical calculation was carried out. The intermediates of the metal center and the conversion of the metal and their site-specific slab models to its corresponding the intermediate state M-OH, M–O, and M–OOH formation are shown (Figs. 4i and S38–S40). The four-electron OER pathway (M* → *OH → *O → *OOH → O₂) shows distinct site-dependent energetics. At metal centers, *OH formation (ΔG₁ = 2.32 eV for Co-site and 2.13 eV for Fe-site) emerges as the rate-limiting step, while at O-sites the *OOH formation penalty (ΔG₃ = 3.01 eV) dominates. The computed η^OER^ (max(ΔG) − 1.23 V) is yielded 1.09 V (Co-site), 0.90 V (Fe-site), and 1.78 V (O-site) (Fig. 4j). The estimated η^OER^ highlighted that the Fe-site is the energetically favorable OER active site, whereas Co-site suffered from the *OH over-stabilization and surface O-site exhibited poor *OOH kinetics. Bader charge analysis confirms substantial electron redistribution upon F incorporation. The adsorbed localized F atom accumulates − 0.46 e, with electron depletion localized primarily on Fe (+ 0.72 e) and to a lesser extent on adjacent Co atoms (+ 0.46 e, + 0.52 e), evidencing strong electron withdrawal and polarization of the metal–O network. The corresponding charge density difference (CDD) reveals complementary spatial features, with electron accumulation (yellow) centered on F and depletion (blue) on neighboring metal centers, consistent with a strong electron-withdrawing effect. This interfacial polarization subtly upshifts Co *d* states relative to Fe, rationalizing stronger Co–adsorbate coupling and stabilizing Fe sites closer to the optimal binding condition (Fig. S41). The combined Bader–CDD–PDOS–ΔG framework therefore establishes a coherent mechanistic picture: Surface F functions as an anion promoter that withdraws electron density, polarizes the M–OH coordination environment, and selectively tunes Fe *d* states to balance HER and OER energetics. The result is a bifunctional active site distribution, where Fe centers are optimized for both near-thermoneutral H binding and reduced OER overpotentials, consistent with the experimentally observed catalytic synergy. Projected densities of states (PDOS) reveal metal-dominated states at the Fermi level (E_f_) with d-band centers ε_*d*_(Co) = − 1.41 eV and ε_*d*_(Fe) = − 1.49 eV, for CoFe LMH-3, whereas the PDOS reveal metal-dominated states at the Fermi level (E_f_) with d-band centers ε_*d*_(Co) = − 1.165 eV and ε_*d*_(Fe) = − 0.945 eV, indicating that Fe E_d_ values lie closer to the Fermi level (E_f_) thermoneutral binding regime for F-CoFe LMH-8 (Fig. 4k–l).

### Corrosion and In Situ Electrochemical Study

**Fig. 5:**
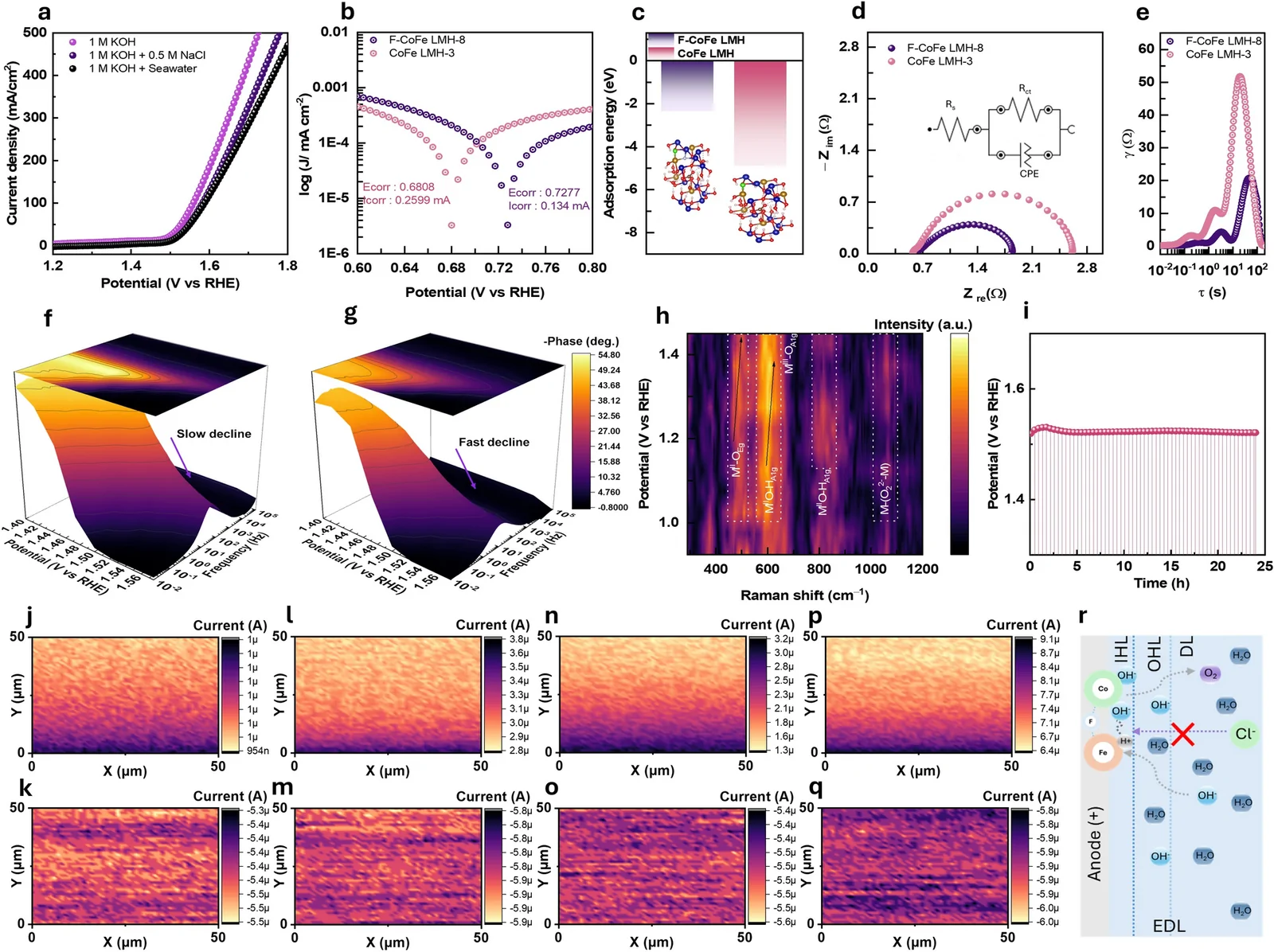
**a** LSV of F-CoFe LMH-8 in 1 M KOH, stimulated seawater (1 M KOH + 0.5 M NaCl) and 1 M KOH in real seawater. **b** Linear polarization curves for CoFe LMH-3 and F-CoFe LMH-8 in stimulated seawater. **c** Cl adsorption energy diagram, **d** Nyquist plot, **e** DRT analysis for CoFe LMH-3 and F-CoFe LMH-8. Multipotential Bode phase analysis for **f** CoFe LMH-3 g F-CoFe LMH-8. **h** In situ Raman analysis and **i** stability for F-CoFe LMH-8 in stimulated seawater. SECM **j** substrate, **k** tip current for CoFe LMH-3 at 0.3 V vs Ag/AgCl, and **l** substrate, **m** tip current at 0.35 V vs Ag/AgCl. The **n** substrate, **o** tip current for F-CoFe LMH-8 at 0.3 V vs Ag/AgCl, and **p** substrate and **q** tip current at 0.35 V vs Ag/AgCl. **r** Schematic representation of ions’ behavior in F-CoFe LMH-8

The LSV of the anode catalyst in 1 M KOH, 1 M KOH + 0.5 M NaCl, and 1 M KOH in seawater (geographical location of seawater collection site is presented in Fig. S42) is shown in Fig. 5a. The catalyst performance decreases in 1 M KOH + 0.5 M NaCl and 1 M KOH in seawater compared to the 1 M KOH electrolyte. The linear polarization is measured to understand the Cl corrosion resistance properties of the catalysts as presented in Fig. 5b. The CoFe LMH-3 showed a corrosion potential (E_corr_) of 0.6808 V with a corrosion current (I_corr_) of 0.2599 mA. The E_corr_ value is shifted higher value 0.7277 V, and the I_corr_ is decreased to 0.134 mA for the F-CoFe LMH-8 catalyst. The positive shift of E_corr_ and lower I_corr_ value indicates that the F-CoFe LMH-8 exhibits higher Cl^−^ corrosion resistance compared to the CoFe LMH-3. To validate the chlorophobic barrier mechanism of F-CoFe LMH-8, DFT calculations were performed comparing Cl^−^ adsorption energies on pure CoFe LMH-3 and F-CoFe LMH-8 surfaces. The adsorption energy of Cl^−^ on pure CoFe LMH was calculated to be − 4.092 eV, indicating strong Cl^−^ affinity for the unprotected surface, which explains the susceptibility of pristine CoFe LMH-3 to chloride corrosion in seawater. In contrast, F-doping on the F-CoFe LMH-8 framework significantly weakened Cl^−^ adsorption to − 2.354 eV, a destabilization of 1.738 eV. This substantial reduction in Cl^−^ binding energy provides direct DFT evidence for the proposed chlorophobic barrier: F^−^ ions modify the electronic structure of Co and Fe active sites, reducing their affinity for Cl^−^ and suppressing chloride corrosion during seawater electrolysis (Fig. 5c). Nyquist plot was analyzed by applying a constant potential of 1.576 V vs RHE to decipher the R_s_ and R_ct_; F-CoFe LMH-8 (0.66 and 1.24 Ω) showed a smaller semicircle compared to CoFe LMH-3 (0.64 and 2.02 Ω) (Fig. 5d). DRT was derived which separated the overlapping processes, and three different elements were uncovered showing a large resistance peak and slower time constant (τ) for CoFe LMH-3; when compared to F-CoFe LMH-8 the absence of these peaks at higher time constants and the smaller resistance peak modulates the reaction site necessary much quickly for F-CoFe LMH-8 for M^III^ –O_A1g_ formation (Fig. 5e). To study the behavioral changes, Bode plot was taken to analyze the change and is presented in Fig. 5f, g. Bode plot shows that there is a reduction in the phase angle, as well as a gradual faster decline in the phase angle for F-CoFe LMH-8 in the lower frequency regions. The peak shift at 1.45 V represents the onset of OER, this peak shift to higher frequency is observed much earlier for F-CoFe LMH-8, and this might be due to formation of faster Helmholtz layer/electrochemical double layer (EDL) near the surface of the catalyst. Furthermore, to analyze the evolved oxygen species, differential electrochemical mass spectrometry (DEMS) was analyzed in 0.5 M NaCl, where mass signal of O for F-CoFe LMH-8 indicated higher than that of CoFe LMH-3, indicating a greater abundance of oxygenated intermediates (Fig. S43). Furthermore, in situ Raman at various potentials was performed to identify the catalytic activity of CoFe LMH-3 and F-CoFe LMH-8 (Figs. 5h and S44). The CoFe LMH-3 shows an E_g_ band (M^II^–O) along with A_1g_ (MO–H) as a result of the metal–oxygen and metal-oxo-hydrogen bond, additionally the in-bound M-OH and M–O^2−^–M bond formations are absent, whereas in F-CoFe LMH-8, the E_g_ is formed, additionally at 550–600 cm^−2^ the M^II^–O_A1g_ band broadens and upshifts signifying the formation M^III^–O_A1g_, and the M^III^ act as active centers for the catalytic reactions. Additionally, a bridging peroxo species (*μ*) is formed. Since the M^II^–O_Eg_ vibrations are consistent and M^II^–O_A1g_ broadens and upshifts, delaying M-OOH transitions can be confirmed (Fig. S45) [22, 44]. Furthermore, ex-situ XPS reveals the potential-dependent surface oxidation dynamics of the catalyst (Fig. S46). Ex-situ XPS analysis reveals that anodic polarization drives dynamic surface reconstruction, progressively oxidizing the Co and Fe environments to transform the pre-catalyst into an active state enriched with the high-valence metal-oxo motifs requisite for O–O bonding sites. Stability of the catalysts was then performed in 1 M KOH + 0.5 M NaCl by applying 50 mA cm^−2^, and a negligible degree of degradation was found (Fig. 5i). Since the catalyst remained stable in stimulated seawater, chronopotentiometric stability was then performed in 1 M KOH + seawater by applying 50 mA cm^−2^, and only negligible degradation was observed after the 24 h of stability (Fig. S47). To analyze the micro-kinetics, in situ SECM was employed by applying 0.3 V vs Ag/AgCl and 0.35 V vs Ag/AgCl. The substrate and tip potential of CoFe LMH-3 at 0.3 V vs Ag/AgCl achieves I_s_ of 0.954 – 1 μA and I_t_ of A and on applying 0.35 V vs Ag/AgCl the current gradually increased to I_s_ of 2.8 – 3.8 μA and I_t_ of − 5.8 – − 5.9 μA (Fig. 5j–m). In contrast, F-CoFe LMH-8 showed better performance onsetting early with a current response of I_s_ of 1.3 – 3.2 μA and corresponding I_t_ of − 5.4 – − 5.6 μA, when bias potential was increased to 0.35 V vs. Ag/AgCl; a corresponding increase of I_s_ of 6.4 – 9.1 μA and I_t_ of − 5.8 – − 6.0 μA was observed (Fig. 5n–q). Corroborating the kinetic and structural insights from the preceding DEMS, in situ Raman, DFT, and Bode DRT analyses, these elevated SECM current responses confirm a highly enriched local OH^−^ environment on the F-CoFe LMH-8. This dense layer of OH^−^ ions sustains stable OER activity and acts as a robust chlorophobic barrier within the EDL, effectively reducing Cl^−^ influx to lower corrosion and enhance long-term durability (Fig. 5r) [35, 40]. FT-IR was analyzed ex situ, and similar to OER, the OH stretching peak was shifted to a higher wavenumber and with lesser intensity which may be created due to the vast number of oxygen vacancy created when potentiometric tests were performed (Fig. S48). Furthermore, the catalyst was then dried and analyzed for XPS and found that a shift was observed in Co 2*p* and Fe 2*p*, whereas M–O bonds increased after stability while analyzing O 1*s* signifying the alterations to the catalyst during OER (Figs. S49–S51) [45].

### Full-Cell Anion Exchange Membrane Water Electrolyzer

**Fig. 6:**
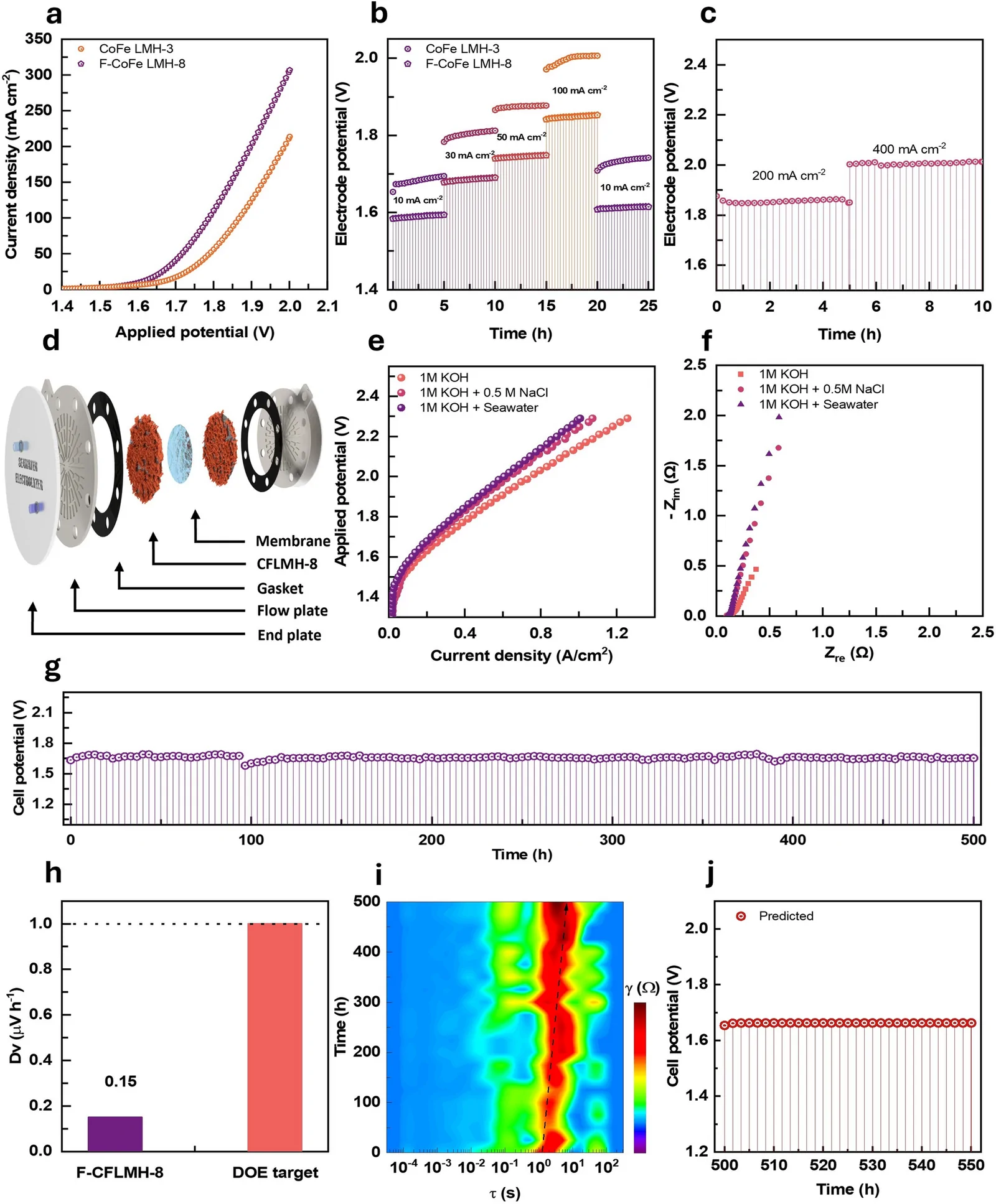
**a** LSV profile and **b** durability study at multi-step current density in 1 M KOH + 0.5 M NaCl of CoFe LMH-3 and F-CoFe LMH-8 catalyst. **c** Stability test of F-CoFe LMH-8 at applying fixed current density of 200 mA cm^−2^ and 400 mA cm^−2^ in 1 M KOH + 0.5 M NaCl. **d** Schematic representation of AEMWE device, **e** polarization plot, and **f** Nyquist plots of bifunctional F-CoFe LMH-8 catalyst integrated AEMWE device in 1 M KOH, 1 M KOH + 0.5 M NaCl, and 1 M KOH + seawater. **g** Stability of AEMWE device, **h** degradation rate comparison to the U.S. DOE target, **i** DRT analysis of full cell over the various time for F-CoFe LMH-8 in 1 M KOH + seawater, **j** predicted stability for F-CoFe LMH-8 with time series analysis

After systematic HER and OER performances evolution under the three-electrode configuration, the F-CoFe LMH-8 is explored for the overall water splitting in a conventional two-electrode system (F-CoFe LMH-8||F-CoFe LMH-8) at room temperature in 1 M KOH + 0.5 M NaCl. Figure 6a shows the F-CoFe LMH-8 || F-CoFe LMH-8 required a voltage of 1.61 V to attain 10 mA cm^−2^. Stability of the catalyst was analyzed by applying various potentials of 10, 30, 50, and 100 mA cm^−2^ for 25 h (Fig. 6b). The catalyst exhibited stable performance during the stability testing without any apparent performance degradation. Thereafter, the stability is also tested by applying a high current density of 200 and 400 mA cm^−2^ to check the robustness of the catalyst, under harsh conditions (Fig. 6c). Notable, the F-CoFe LMH-8||F-CoFe LMH-8 system exhibited stable performance at higher current density, suggesting its robust nature in the presence of corrosive Cl^−^ ions. Moreover, ClER is evaluated using N, N-diethyl-p-phenylenediamine (DPD) colorimetric test. The electrolyte is collected after chronopotentiometry measurements at fixed current density of 10, 30, 50, and 100 mA cm^−2^ for 5 h. The colorimetric reaction of hypochlorite and N, N-diethyl-p-phenylenediamine (DPD) is presented in Figs. S52–S54. The UV–Vis spectra of the DPD solution are presented in Fig. S55. The intensity of spectra is directly correlated with the hypochlorite ions concentration, which is formed due the ClER. Notably, week UV–Vis spectral intensity for the F-CoFe LMH-8 catalyst indicating very low hypochlorite ions formations at different current densities. The F-CoFe LMH-8 || F-CoFe LMH-8 is subjected to the multi-step chronopotentiometry in 1 M KOH + seawater to analyze the robustness of the catalyst in real-seawater conditions (Fig. S56). The CoFe LMH-8 catalyst exhibited stable performance throughout the entire multi-step current stability test. Moreover, near identical LSV profile measured before and after the stability test suggested the excellent durability of the CoFe LMH-8 catalyst in real seawater (Fig. S57).

In regard, to the excellent bifunctional performances in seawater under the conventional two-electrode configuration, F-CoFe LMH-8 is tested as bifunctional catalyst to be fabricated as an anion exchange membrane water electrolyzer (AEMWE). The schematic presentation of the AEMWE is shown in Figs. 6d and S58. The F-CoFe LMH-8 catalyst-coated electrodes were sandwiched between a Fumasep FAS 50 membrane, and the cross-sectional image of the membrane electrode assembly (MEA) is presented in Fig. S59. The comparative performance of the F-CoFe LMH-8 and CoFe LMH-3 catalyst in 1 M KOH at 50 °C is presented in Fig. S60. The F-CoFe LMH-8 catalyst delivered 1 A cm⁻^2^ at 2.14 V, outperforming the undoped CoFe LMH-3 catalyst which required 2.3 V to attained 1 A cm^−2^. The F-CoFe LMH-8 bifunctional catalyst integrated AEMWE is tested in series of electrolytes of 1 M KOH, 1 M KOH + NaCl, 1 M KOH + Seawater at 50 °C as presented in Fig. 6e. The device required 2.14, 2.23, and 2.29 V to achieve 1 A cm^−2^ in 1 M KOH, 1 M KOH + 0.5 M NaCl, and 1 A cm^−2^ in 1 M KOH, respectively, indicating that the device polarization profile or performance is almost preserved in the presence of seawater. Moreover, EIS of the AEMWE is performance in the presence of these different electrolytes. The Nyquist measurements were tested by applying 0 V bis potential, which suggests an increase in interfacial charge transfer kinetics due to chlorine incorporation, consistent with the corresponding LSV (Fig. 6f). To analyze the durability of the device in stimulated seawater, the system MEA was fabricated for F-CoFe LMH-8 and commercial NiFe LDH. The commercial electrode degraded within 40 h, while a current density of 0.125 A cm^−2^ was applied, whereas F-CoFe LMH-8 (+ /−) showed excellent durability without any degradation at a current density of 0.5 A cm^−2^ (Figs. S61 and S62). The anolytes were then collected and analyzed with inductively coupled plasma mass spectrometry (ICP-MS), which showed substantial leaching of the commercial catalyst, whereas for F-CoFe LMH-8 the leaching was negligible (Co of 0.005 ppm and Fe with 0.98 ppm, Fig. S63). Furthermore, the AEMWE device stability was analyzed in 1 M KOH + seawater, which exhibited device steady performance without loss for 500 h, indicating excellent durability of the F-CoFe LMH-8 catalyst in the seawater electrolyte (Fig. 6g). As the stability of the catalyst in key factor for the practical feasibility of seawater electrolysis, especially high halide ions concentration, the degradation rate (Dv) was calculated, which was found to be 0.15 uV h^−1^ which is significantly lower than department of energy (DOE) target of 1 uV h^−1^ (Fig. 6h). EIS monitoring over the operational time (Fig. S64) revealed a slight enlargement of the Nyquist semicircle, consistent with minimal degradations observed in long‑term MEA operation. Complementary DRT analysis resolved the underlying processes, highlighting well‑maintained cathodic and anodic distributions (Fig. 5i) [46]. The machine learning and deep learning techniques are becoming increasingly important in materials and device research [47]. In this case, the stability of F-CoFe LMH-8 was forecasted and predicted using a machine learning-based long short-term memory (LSTM) technique. The experimental and forecasted voltage values are shown in Fig. S65. The experimental and forecasted values are closely associated, and no usual deviation was observed. Furthermore, the model accurately predicts the voltage of CoFe LMH-8 over the next 50 h, as shown in Fig. 6j. This suggests that the LSTM is an excellent machine learning technique for predicting and forecasting the stability of CoFe LMH-8. The cross-sectional SEM of the MEA was then analyzed to visualize the membrane electrode interface, which remained intact after 500 h of stability in seawater (Fig. S66). The MEA was subsequently dismantled, and the individual half-electrodes (cathode/anode) were examined using SEM, elemental mapping, EDS. These analyses revealed the presence of majority elements like Fe and Co along with some trace elements such as calcium and chlorine, albeit in very minor quantities (Figs. S67 and S68, Tables S5 and S6).

The performance of the F-CoFe LMH-8 catalyst was compared with previously published literature, as presented in Table S4. Furthermore, a gram-scale synthesis protocol is developed as a heuristic validation of the scalable synthesis protocol as industrial electrolyzer large quantity of catalyst (Fig. S69a). The physicochemical characterization of bulk-synthesized catalyst is analyzed for the morphological and crystallographic structure by XRD, Raman analysis, and FE-SEM with EDS and elemental mapping. The physicochemical characterization confirmed that the bulk-synthesized F-FeCo LMH catalyst’s crystallographic phase and morphology is well matched with the batch synthesized catalyst as presented in Fig. S69b-l. Furthermore, the electrochemical performance of the bulk-synthesized catalyst was analyzed against that of the bench-scale synthesis (Fig. S70). Both catalysts exhibited comparable activity in the anodic and cathodic half-cell tests. Furthermore, EIS measurements for the OER in 1 M KOH + 0.5 M NaCl revealed that similarity remained invariant, suggesting that the MgO-mediated synthesis methodology can be effectively upscaled, while retaining the nanosheet-like morphology, enabling large-scale production without any significant losses in catalytic activity or electrochemical performance. Additionally, in alignment with the green initiative, the electrolyzer cell of F-CoF LMH-8( −) || F-CoF LMH-8 ( +) AEMSWE was integrated with solar PV cell to demonstrate the practical application of the developed system (Fig. S71, Video S1).

The fluorine‑mediated tuning of CoFe‑LMH induces a pronounced modulation of the Fe–O coordination environment, generating a higher population of high‑spin Fe active sites, as evidenced by EPR spectroscopy and WT‑EXAFS analysis without altering the Co metal active centers. Despite the incorporation of fluorine, the layered nanosheet-like structure of F‑CoFe‑LMH was well preserved exhibiting lattice expansion attributed to the altered electronic environment around Fe centers. The increased density of catalytically accessible high‑spin sites directly enhances the intrinsic activity toward both HER and OER.

Moreover, the formation of the M^III^-O A_1g_ band in the F‑CoFe LMH provides an additional protective effect, effectively mitigating chlorine‑induced corrosion under seawater electrolysis conditions. The strategic exploitation of spin‑state modulation through fluorine incorporation significantly optimizes the electronic structure of the catalyst, promoting accelerated charge transfer kinetics and facilitating rapid interfacial electron migration at the electrode–electrolyte boundary while effectively blocking the Cl ion attack at the electrode–electrolyte interface. Collectively, these synergistic effects culminate in superior seawater electrolysis performance with minimal structural degradation and enhanced long‑term operational stability.

## Conclusions

To summarize, a robust and stable bifunctional electrocatalyst catalyst is engineered for sustained seawater electrolysis incorporating F-doping in CoFe LMH. The precision tuning of CoFe LMH by F-doping manipulated Fe coordination site without altering the Co OER active metal centers. The as-prepared F-CoFe LMH-8 showed excellent HER and OER performance, achieving 81.23 and 265.5 mV @ 10 mA cm^−2^, respectively. F-doping manipulated Fe sites for higher Fe–O bonding, which resulted in sustained high OH- ions influx at EDL resulting in enhanced Cl corrosion inhibition, enhancing performance and durability. Besides, the AEM seawater electrolyzer F-CoFe LMH-8 ||F-CoFe LMH-8 achieves 1 A cm^−2^ @ 2.3 V running stably for 500 h at 0.125 A cm^−2^ applied current, with a remarkably low degradation rate of 0.15 uV h^−1^ demonstrating the industrial viability and surpassing the DOE technical targets. This work provides a new paradigm for facile synthesis for high-performance, corrosion-resistant seawater catalyst for sustained AEM seawater electrolyzer operations.

## Supplementary Information

Below is the link to the electronic supplementary material.

Supplementary file1 (DOCX 8334 kb)

Supplementary file2 (MP4 9585 kb)
