# Correction: High Polarity Doping of CoFe Layered Hydroxides: Bifunctional and Corrosion-Resistant Anion Exchange Membrane Seawater Electrolyzers

**DOI:** 10.1007/s40820-026-02303-8

**Published:** 2026-07-21

**Authors:** Anandhan Ayyappan Saj, Sampath Prabhakaran, Mohsin Rasool, Kousik Bhunia, Dongho Lee, Hyunseok Ko, Tukaram D. Dongale, Muthukumar Perumalsamy, Arul Saravanan Raaju Sundhar, Do Hwan Kim, Sang Jae Kim

**Affiliations:** 1 Nanomaterials & System Lab, Major of Mechatronics Engineering, Faculty of Applied Energy System, Jeju National University, Jeju, 63243 South Korea; 2 Nanomaterials & System Lab, Major of Mechanical System Engineering, College of Engineering, Jeju National University, Jeju, 63243 South Korea; 3 Green Hydrogen Glocal Leading Research Center (gH2-RC), Jeju National University, Jeju, 63243 Republic of Korea; 4 Research Institute of New Energy Industry (RINEI), Jeju National University, Jeju, 63243 South Korea; 5 Division of AI Convergence Research, Korea Institute of Ceramic Engineering and Technology (KICET), Jinju, 52851 Republic of Korea; 6 University of Ulsan, Ulsan, 44776 Republic of Korea; 7 Division of Science Education and Institute of Fusion Science, Department of Energy Storage/Conversion Engineering (BK21 FOUR), Jeonbuk National University, Jeonju, Jeonbuk 54896 Republic of Korea; 8 Computational Electronics and Nanoscience Research Laboratory, School of Nanoscience and Biotechnology, Shivaji University, Kolhapur, Maharashtra 416004 India

**Correction to: Nano-Micro Lett. (2026) 18:393** 10.1007/s40820-026-02230-8

Following publication of the original article [1], the authors reported that Fig. 3 needed to be updated.

The correct Fig. 3 has been provided in this Correction.

The incorrect Fig. 3 is:

**Figure:**
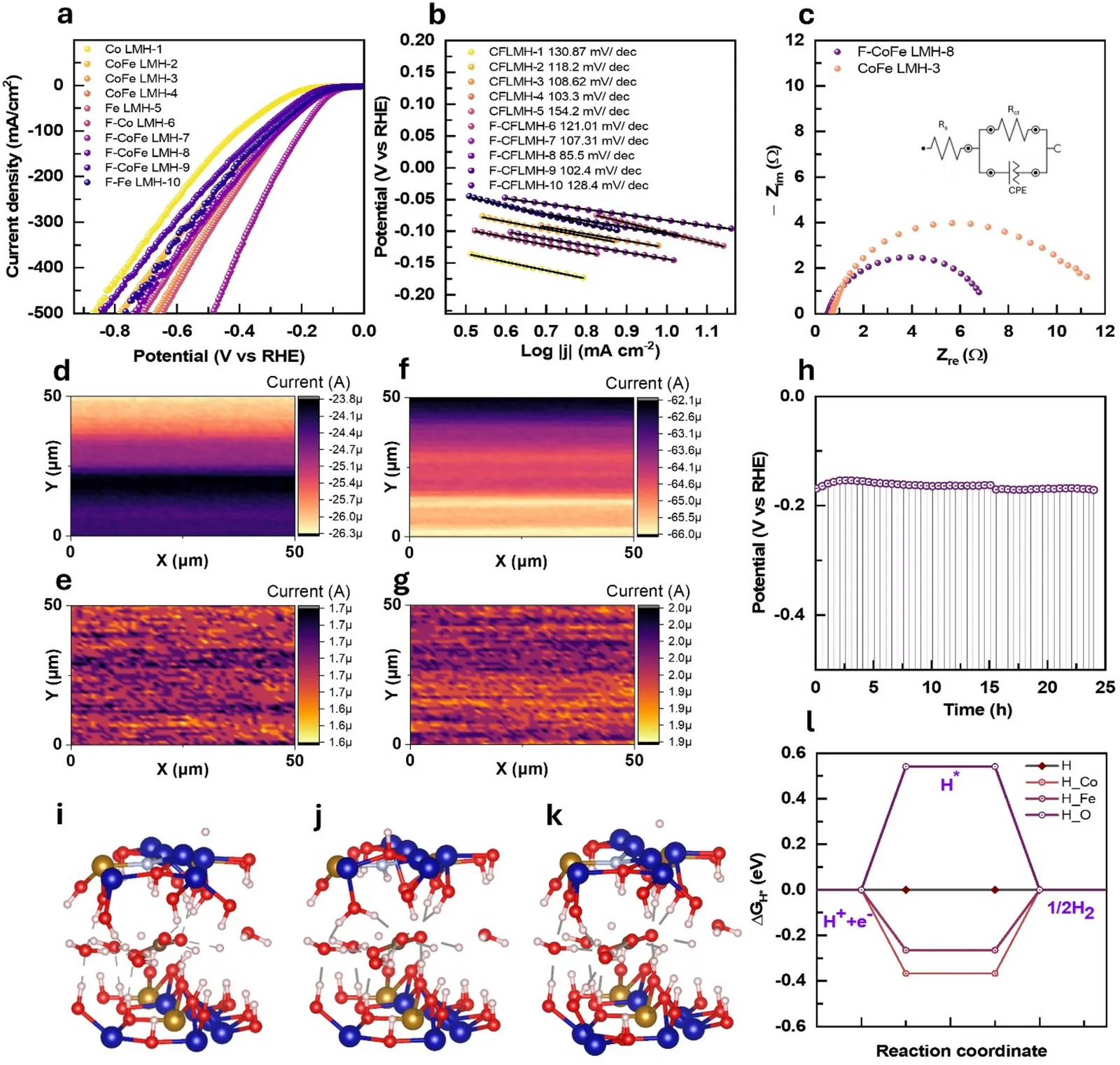

Fig. 3 ** a** LSV profile and **b** Tafel slope of the different composition F-doped and undoped FeCo LMH catalyst. **c** Nyquist plot of CoFe LMH-3 and F-CoFe LMH-8. SECM analysis of CoFe LMH-3 catalyst **d** surface and e tip current profile. SECM analysis of F-CoFe LMH-8 catalyst **f** surface and **g** tip current profile. h Electrochemical stability of F-CoFe LMH-8 at − 50 mA cm^−2^ constant current density. H* adsorption energy at **i** Co-site,** j** Fe-site, and **k** O-site of F-doped FeCo LMH catalyst. **l** H* adsorption Gibbs free energy on Co, Fe, and O-sites.

The correct Fig. 3 is:

**Figure:**
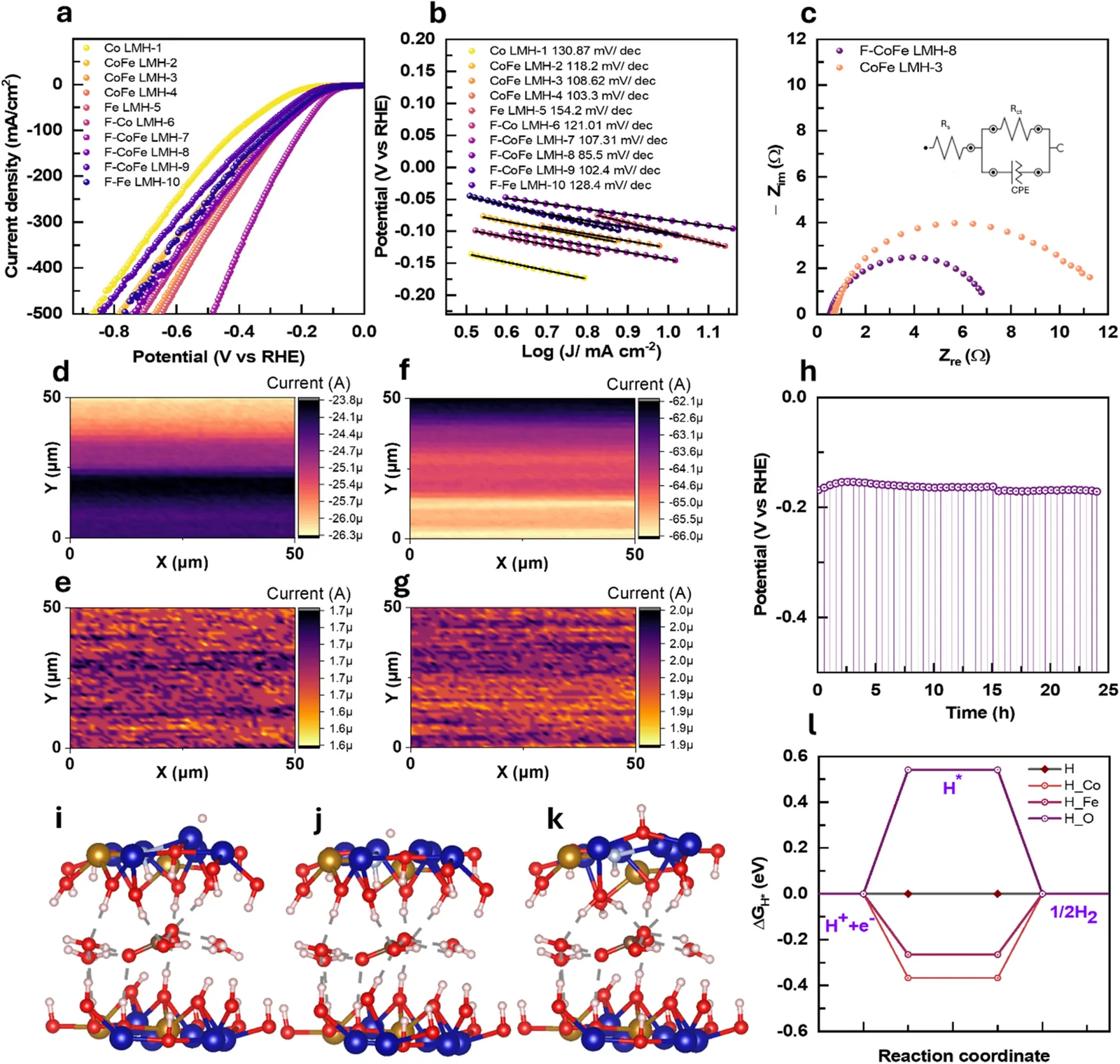

Fig. 3 ** a** LSV profile and **b** Tafel slope of the different composition F-doped and undoped FeCo LMH catalyst. **c** Nyquist plot of CoFe LMH-3 and F-CoFe LMH-8. SECM analysis of CoFe LMH-3 catalyst **d** surface and e tip current profile. SECM analysis of F-CoFe LMH-8 catalyst **f** surface and **g** tip current profile. h Electrochemical stability of F-CoFe LMH-8 at − 50 mA cm^−2^ constant current density. H* adsorption energy at **i** Co-site,** j** Fe-site, and **k** O-site of F-doped FeCo LMH catalyst. **l** H* adsorption Gibbs free energy on Co, Fe, and O-sites.

The original article [1] has been corrected.
