# Supplementary material for: High Polarity Doping of CoFe Layered Hydroxides: Bifunctional and Corrosion-Resistant Anion Exchange Membrane Seawater Electrolyzers
Source: Nanomicro Lett. 2026 Jun 1;18:393. doi: 10.1007/s40820-026-02230-8 (PMC13226751; doi:10.1007/s40820-026-02230-8)
Supplement: Supplementary file 1 — Supplementary file1 (DOCX 8334 kb) [file 40820_2026_2230_MOESM1_ESM.docx]

Supporting Information for

**High Polarity Doping of CoFe Layered Hydroxides: Bifunctional and Corrosion Resistant Anion Exchange Membrane Seawater Electrolyzers**

Anandhan Ayyappan Saj^1^, Sampath Prabhakaran^5^, Mohsin Rasool^6^, Kousik Bhunia^1^, Dongho Lee^6^, Hyunseok Ko^5^, Tukaram D Dongale^8^, Muthukumar Perumalsamy^1^, Arul Saravanan Raaju Sundhar^1^, Do Hwan Kim^7*^, Sang Jae Kim^1,2,3,4*^

^1^ Nanomaterials & System Lab, Major of Mechatronics Engineering, Faculty of Applied Energy System, Jeju National University, Jeju, 63243, South Korea

^2^ Nanomaterials & System Lab, Major of Mechanical System Engineering, College of Engineering, Jeju National University, Jeju, 63243, South Korea

^3^ Green Hydrogen Glocal Leading Research Center (gH_2_-RC), Jeju National University, Jeju 63243, Republic of Korea

^4^ Research Institute of New Energy Industry (RINEI), Jeju National University, Jeju, 63243, South Korea

^5^ Division of AI Convergence Research, Korea Institute of Ceramic Engineering and Technology (KICET), Jinju, 52851, Republic of Korea

^6^ University of Ulsan, Ulsan 44776, Republic of Korea

^7^ Division of Science Education and Institute of Fusion Science, Department of Energy Storage/Conversion Engineering (BK21 FOUR), Jeonbuk National University, Jeonju, Jeonbuk, 54896, Republic of Korea

^8^ Comutational Electronics and Nanoscience Research Laboratory, School of Nanoscience and Biotechnology, Shivaji University, Kolhapur,416004, Maharashtra, India

^*^Corresponding author. E-mail: [kimsangj@jejunu.ac.kr](mailto:kimsangj@jejunu.ac.kr) (Sang Jae Kim); [dhk201@jbnu.ac.kr](mailto:dhk201@jbnu.ac.kr) (Do Hwan Kim)

**S1 Supplementary Detailed Procedures**

**S1.1 Materials**

Cobalt chloride hexahydrate (CoCl_2_·6H_2_O), ammonium fluoride (NH_4_F), iron sulfate hexahydrate (FeSO_4_⋅7H_2_O), potassium hydroxide (KOH), acetone (CH_3_COCH_3_), and ethanol (C_2_H_6_OH) were purchased from Dae Jung Chemicals and Metals Pvt. Ltd., South Korea. Nickel foam (NF) (1 mm, 110 PPI) was purchased from Heze Jiaotong Group, China. Deionized water (DI) is used throughout the experiments. All the chemicals are used as received without any further purification. Seawater was collected from Jungmun Beach, Seogwipo-si, Jeju-do, South Korea

**S1.2 Material characterization**

Field-emission scanning electron microscopy (FE-SEM) in conjunction with energy-dispersive X-ray spectroscopy (MIRA3, TESCAN, Czechia) and JEOL JEM-2100F high-resolution transmission electron microscopy (HRTEM) at KBSI (Korea Basic Science Institute) is used to investigate the microstructure of the synthesized catalyst. The crystalline structure and phase of the synthesized catalysts were analyzed by powder X-ray diffraction (XRD) method using Malvern Panalytical (Empyrean) with Cu-Kα radiation (λ = 1.5418 Å) at a scan rate of 5^o^/min from 5-90^o^. The surface chemical state is analyzed by an X-ray photoelectron spectrometer (Theta Probe Angle-Resolved X-ray Photoelectron Spectrometer System, Thermo Fisher Scientific). Raman analysis was carried out using Thermo Scientific™ DXR3xi Raman Imaging Microscope. In-situ Raman studied in Horiba LABram HR Evolution. XAS analysis performed in Pohang Accelerator Laboratory. Fourier Transform Infrared (FT-IR) spectroscopy measurement is carried out using Bruker Alpha II. Electron paramagnetic resonance measured through CIQTEK EPR200M. Superconducting quantum interference device - vibrating sample magnetometer (SQUID-VSM) was analyzed by MPMS3-Quantum design. Nuclear magnetic resonance (NMR) spectroscopy was analyzed with JEOL 400 MHz with CPMAS (4mm od) NMR spectrometer. Inductively coupled plasma mass spectrometry (ICP-MS) was carried out with ICP-MS; iCAP RQ, Thermo Fisher Scientific.

**S1.3 Reaction mechanism**

The proposed fabrication route is inherently scalable and well‑suited for the large‑volume synthesis of nanocatalysts. During the initial hydration and dissolution steps, MgO undergoes surface hydroxylation followed by progressive ion release, ultimately leading to the formation of Mg(OH)_2_, Mg is replaced by Co/Fe leading to the formation of LDH catalyst.

The overall transformation can be represented as:

${MgO}_{(S)}+H_{2}O\to MgOH+{{OH}^{-}}_{(surface)}\to{{Mg}^{2+}}_{(aq)}+{2{OH}^{-}}_{(aq)}\to{Mg(OH)}_{2}$ (S1)

${{Mg(OH)}_{2}}_{(S)}+{Co}^{2+}/{Fe}^{2+}+H_{2}O\to\left( \alpha\right){{CoFe(OH)}_{2}}_{(s)}+ {{Mg}^{2+}}_{(aq)}$ (S2)

**S2 Electrochemical mesurements**

**S2.1 Electrochemical half-cell measurements**

All of the electrochemical measurements were carried out using an Autolab PGSTAT302N electrochemical workstation. The individual electrochemical properties of the catalyst were investigated by a conventional three-electrode configuration where the catalyst was coated on nickel foam (1 cm^2^), which is used as the working electrode, Hg/HgCl_2_ is used as the reference electrode, and graphite sheet (1 cm^2^) is used as the counter electrode, respectively. The linear sweep voltammetry (LSV) was performed at a 5 mV s^−1^ scan rate in 1M KOH solution. Electrochemical impedance spectroscopy (EIS) is used to analyze charge transfer kinetics across the electrode/electrolyte interfaces. Chronopotentiometry is used to identify the stability of the prepared catalysts by applying a current density of 50 mA cm^−2^. All the measured potentials against the Hg/HgCl_2_ electrode were converted into the reversible hydrogen electrode (RHE) potential by using equation (S3).

$E_{RHE}=E_{\left( Hg/Hg{Cl}_{2} \right)}+0.059 pH+{E^{^{\circ}}}_{\left( Hg/Hg{Cl}_{2} \right)}$ (S3)

The double-layered capacitance (C_dl_) was derived from the CV tests performed at scan rates from 5 to 100 mVs^-1^ to evaluate the electrochemically active surface area. All the electrochemical data were presented without *IR correction*. The electrochemical active surface area (ECSA) was calculated according to equation (S4).

$ECSA = Cdl/C_{s}$ (S4)

Where Cs is found to be 0.04 mF cm ^−2^ under alkaline conditions.

**S2.3 Scanning electrochemical microscopy measurements**

Sensolytics instrument (SECM087) along with Autolab-PGSTAT-302N was used to carry out the SECM measurements with platinum microelectrode with 10 μm diameter. The cell consisted of CoFe LMH-3 and F-CoFe LMH-8 placed at the bottom of the cell, whereas graphite rod and Ag/AgCl were used as counter and reference electrodes, respectively. The electrochemical measurements were performed in 1M KOH for OER and 1M KOH + 0.5 M NaCl. The surface profile was collected at a scan rate of 5 μm s^-1^ in a scan area of 50 × 50 μm. All the measured potentials against the Ag/AgCl electrode were converted into the RHE potential by using equation (S5).

$E_{RHE}=E_{\left( Ag/AgCl \right)}+0.059 pH+{E^{^{\circ}}}_{\left( Ag/AgCl \right)}$ (S5)

**S2.3 In-situ Raman measurements**

In-situ Raman measurements were analyzed through a custom cell where a glassy carbon electrode was coated with the catalyst material using drop casting which was used as the working electrode. A graphite rod was used as the counter electrode and a Hg/HgO reference electrode was used. All the measured potentials against the Hg/HgCl_2_ electrode were converted into the RHE potential by using equation (6).

$E_{RHE}=E_{\left( Hg/HgO \right)}+0.059 pH+{E^{^{\circ}}}_{\left( Hg/HgO \right)}$ (S6)

**S2.4 Distribution of relaxation times (DRT) analysis**

DRT transforms impedance spectra from the frequency domain into time‑domain relaxation time distributions, allowing direct identification and differentiation of dominant and secondary electrochemical reaction processes. Given that polarization impedance is constant and EIS measurements use logarithmically spaced frequencies, the DRT‑reconstructed impedance can be written as described by equation (S7) [S1–S3].

$Z_{DRT}(f)=R_{\infty}\int_{-\infty}^{+\infty} \frac{\gamma(ln \tau)}{1+i2\pi f\tau}d ln \tau$ (S7)

Where Z_DRT_ (f) represents the total impedance of the electrochemical system, R_∞_ represents the ohmic impedance where frequency is infinite, τ is the characteristic relaxation time, f and  is the frequency. DRT is analyzed based on the framework algorithm that optimizes the frequency factor for a DRT-based EIS reconstruction method.

**S2.5 Differential electrochemical mass spectroscopy (DEMS)**

For measuring in-situ DEMS, Type A DEMS cell is used to analyze the extent of O signals in pH 7 in 0.5 M NaCl. Catalysts were coated on graphite rod electrodes (5 mm diameter). The DEMS cell was prepared with catalyst-coated graphite rod as a working electrode, Pt wire as a counter electrode, and screw-type Ag/AgCl (3M KCl) as a reference electrode, respectively. After cell assembly a total of 30 mL was flowed inside the cell after which flow rate was 0.1 mL min^–1^ during analysis.

**S2.6 Anion exchange membrane water electrolyzer (AEMWE)**

The AEMWE was prepared by sandwiching FUMASEP FAS 50 between F-CoFe LMH (cathode) and F-CoFe LMH (anode). Commercial AEMWE was fabricared by sandwiching FUMACEP FAS 50 between Pt/C (cathode) and NiFe LDH (anode). Subsequently, electrochemical experiments were conducted by circulating 1 M KOH + seawater using a peristaltic pump with a feed of 5 mL min^−1^ at room temperatures. The steady-state linear sweep voltammetry measurements were carried out using Arbin Instruments (USA). The stability measurements were carried out in the Neware tester with same flow rate of 5 mL min^-1^ for 125 mA/cm^2^ and 10 mL min^−1^ for 500 mA/cm^2^. Device EIS was carried out in room temperature with flow rate of 5 mL min^−1^ with VIONIC Metrohm potentiostat/galvanostat.

**S3 Density functional theory methodology**

The electrocatalyst was assessed computationally by spin-polarized DFT using the Vienna Ab initio Simulation Package (VASP), with structural models guided by the XRD analysis [S4, S5]. Exchange–correlation effects were treated within the GGA-PBE framework augmented by Grimme’s D3 dispersion correction [S6], and core–valence interactions were described by the projector augmented-wave (PAW) method as implemented in VASP [S7]. To model exposed surfaces, periodic F@CoFe-LMH slab geometries were constructed and separated from their periodic images by sufficient vacuum; cell dimensions were chosen to eliminate spurious inter-slab interactions in all directions. These models were then used to compute projected densities of states (PDOS) and adsorption energies of key intermediates. A 500eV plane-wave cutoff was applied, and structures were optimized until the maximum Hellmann–Feynman force was below 0.02 eV Å⁻¹. For electronic-structure and DOS calculations, Monkhorst–Pack meshes corresponding to a reciprocal-space spacing of ≈0.02 Å⁻¹ were generated with VASPKIT and VASP [S8].

**S3.1 Band center calculation**

The calculation of the *p*- and *d*-band center has been extensively employed for elucidating catalytic behavior [S9, S10]. The determination of the *p* and *d* band center involved the following calculations:

| $\varepsilon=\frac{\int_{-\infty}^{\infty} E\rho(E)dE}{\int_{-\infty}^{\infty} \rho(E)dE}$ | (S8) |
| --- | --- |

where $\rho$ is the projected DOS and *E* is the energy of *p-* orbitals of Fe, Co atoms and *d-* orbitals of C, F, O, H.

**S3.2 HER**

In general, the Gibbs energy change of hydrogen adsorption (ΔG_H*_) on the surface of the catalyst is widely used to determine the HER activity. The catalytic performance was estimated by free energy change during ΔG_H*_ based on the formula proposed in equation (S9) [S11]:

${\Delta G}_{H*} = E_{H*/surf} - E_{surf} - \frac{E_{H_{2}}}{2} + {\Delta E}_{ZPE} - T\Delta S$ (S9)

where E_H*/surf_ -, Esurf, E_H2_, ΔE_ZPE_, and ΔS represent total energies of the slab with H*, the clean surface, the isolated hydrogen molecule, zero-point energy change, entropy change, and temperature (T) taken under standard conditions (298.15K), respectively.

**S3.3 OER**

To compute the OER, a simplified four-electron pathway was adopted to represent the OER mechanism. This process can be described through the following sequence of reactions:

${OH}^{-}+ * \to{OH}^{*} + e^{-}$ ∆G_1_ (S10)

${OH}^{*} + {OH}^{-} \to O^{*}+ H_{2}O + e^{-}$ ∆G_2_ (S11)

$O^{*} + {OH}^{-} \to{OOH}^{*} + e^{-}$ ∆G_3_ (S12)

${OOH}^{*} + {OH}^{-} \to O_{2} + * + H_{2}O + e^{-}$∆G_4_ (S13)

where OH*, O*, and OOH* represent adsorbed intermediates on the catalyst’s active sites, and ∆G₁, ∆G₂, ∆G₃, and ∆G₄ indicate the Gibbs free energy changes for each step.

The Gibbs free energy variation (ΔG) for each reaction step was evaluated using the expression:

$\Delta G = \Delta_{E} + \Delta_{ZPE} - T_{\Delta S} - neU + \Delta G(pH)$ (S14)

where ΔE is derived from the energy of the catalyst with adsorbed oxygen species, Δ_ZPE_ and Δ_S_ represent the differences in zero-point energy and entropy, respectively, and T is the temperature set at 298.15 K (standard conditions, p = 1 bar). The term −*n*eU accounts for the potential bias due to electron transfer, with U representing the electrode potential and n the number of electrons. The pH-dependent correction, ΔG(pH) = Δ*k*T ln([H^+^]), adjusts the free energy based on proton concentration.

Theoretical overpotentials (η) for all investigated structures were determined using the relation:

$\eta_{\mathrm{OER}}=\frac{\max\left\{ {\Delta G}_{1}, {\Delta G}_{2}, {\Delta G}_{3}, {\Delta G}_{4} \right\}}{e}-1.23\left( V \right)$ (S15)

where max {∆G₁, ∆G₂, ∆G₃, ∆G₄} identifies the largest Gibbs free energy change among the elementary steps, and 1.23 V is the standard OER potential. This approach aligns with established methodologies [S12, S13].

The adsorption energy (*E_ads_*) of Cl is calculated as in the following equation:

$E_{ads}=E_{molecule+surface}-(E_{surface}+E_{molecule}$ (S16)

Where *E_surface_* is the energy of the CoFe LMH or F-CoFe LMH  _surface_, *E_molecule_* is the energy of a Cl atom, which is obtained by averaging the total energy of a Cl_2_ molecule. *E_molecule+surface_* represents the total energy of the adsorbed system.

**S3 Supplementary Figures**

**
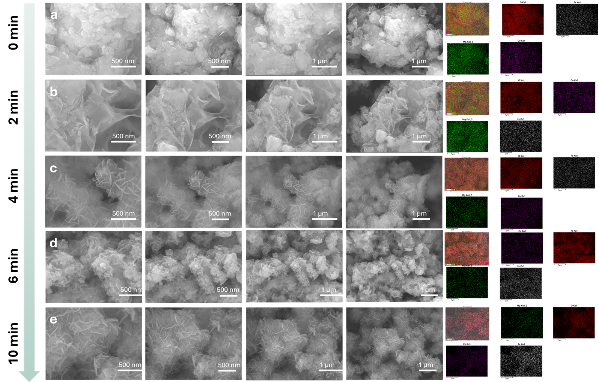
**

**Fig. S1** FE-SEM and elemental mapping at various magnifications of CoFe LMH at various times intervals during catalyst synthesis.

**Fig. S2** P-XRD at various magnifications of CoFe LMH at various times intervals during catalyst synthesis.


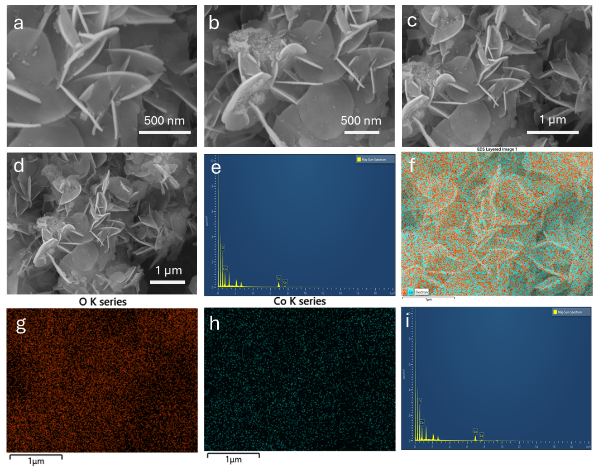


**Fig. S3** FE-SEM at various magnifications of CoFe LMH-1, elemental mapping and EDS spectra.


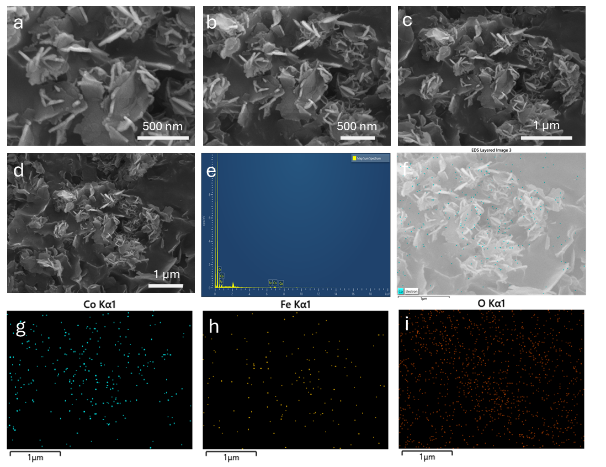


**Fig. S4** FE-SEM at various magnifications of CoFe LMH-2, elemental mapping and EDS spectra.

**
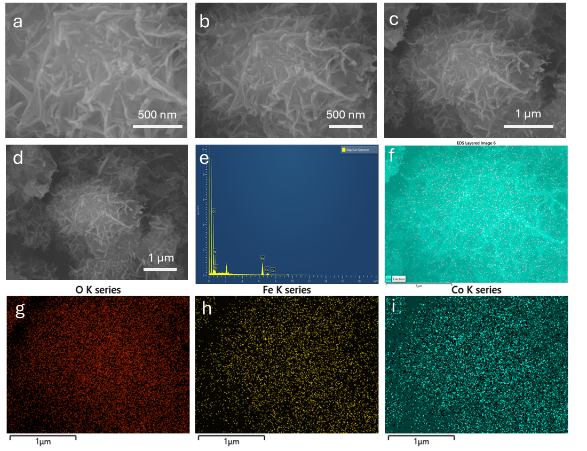
**

**Fig. S5** FE-SEM at various magnifications of CoFe LMH-3, elemental mapping.


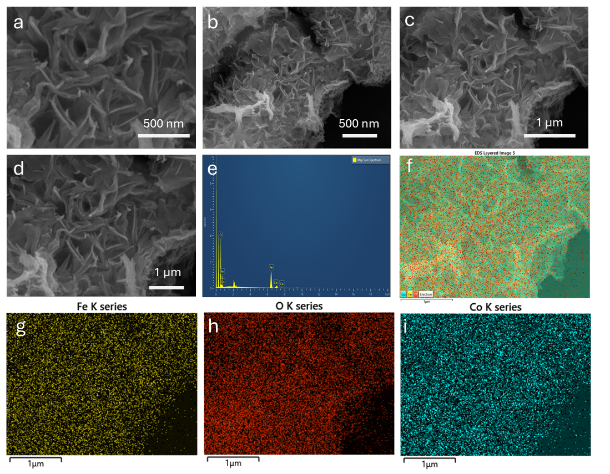


**Fig. S6** FE-SEM at various magnifications of CoFe LMH-4, elemental mapping and EDS spectra.


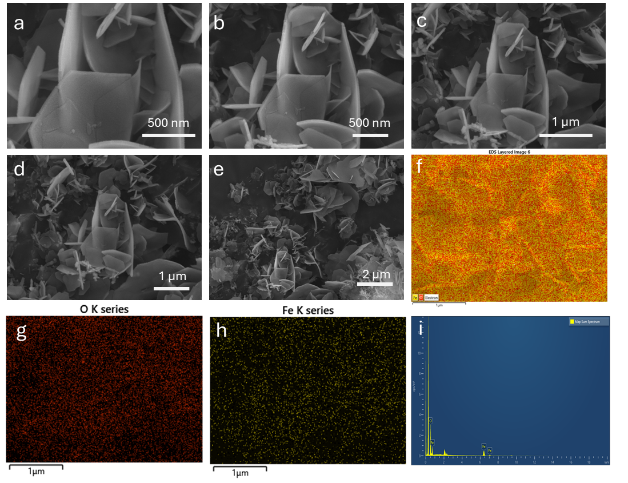


**Fig. S7** FE-SEM at various magnifications of CoFe LMH-5, elemental mapping and EDS spectra.


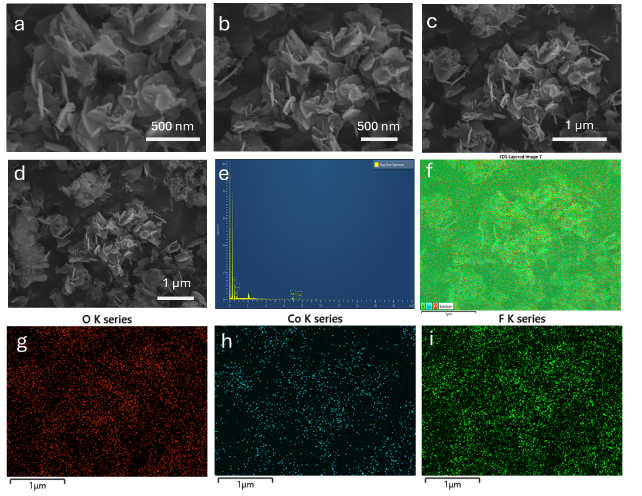


**Fig. S8** FE-SEM at various magnifications of F-CoFe LMH-6, elemental mapping and EDS spectra.


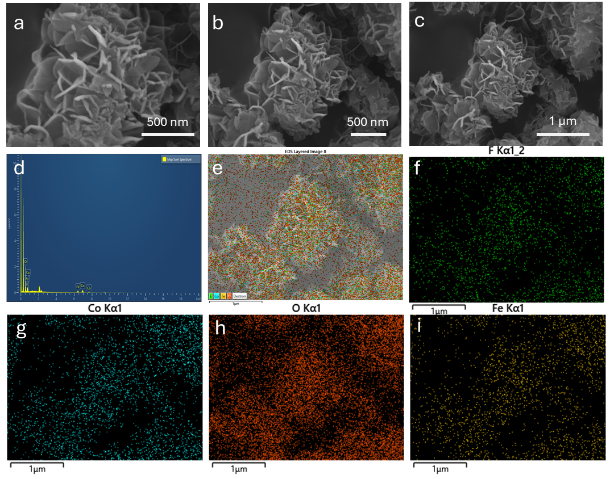


**Fig. S9** FE-SEM at various magnifications of F-CoFe LMH-7, elemental mapping and EDS spectra.


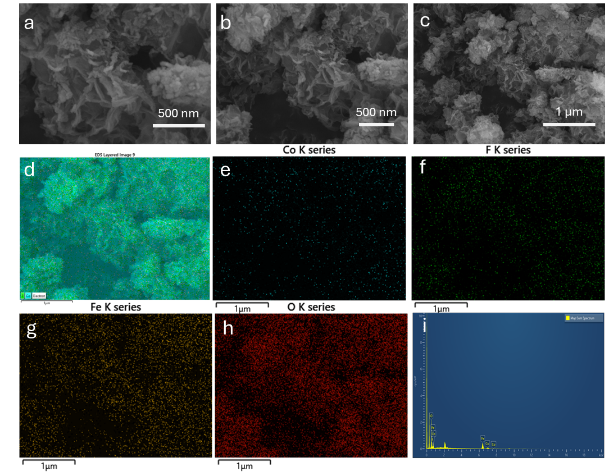


**Fig. S10** FE-SEM at various magnifications of F-CoFe LMH-8, elemental mapping and EDS spectra.


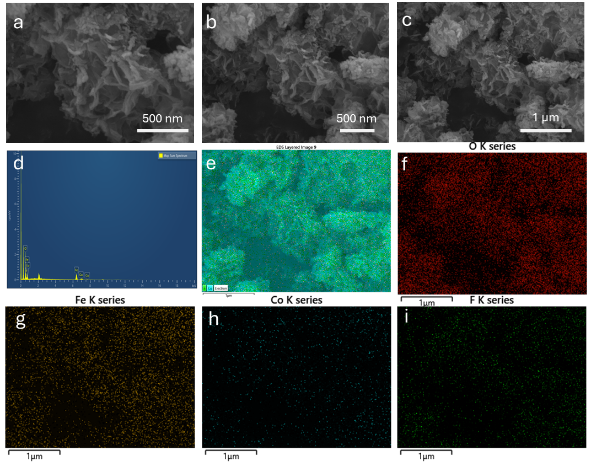


**Fig. S11:** FE-SEM at various magnifications of F-CoFe LMH-9, elemental mapping and EDS spectra.


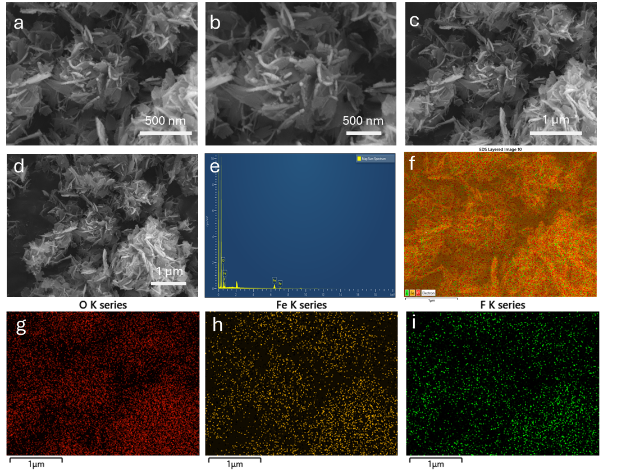


**Fig. S12** FE-SEM at various magnifications of F-CoFe LMH-10, elemental mapping and EDS spectra.


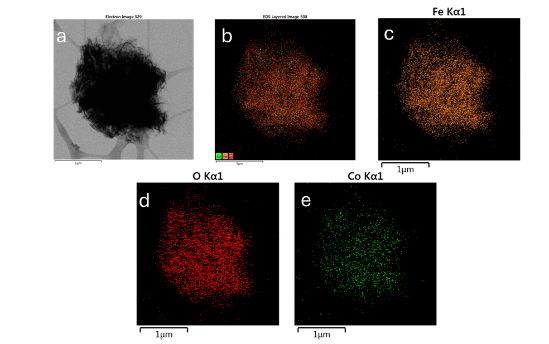


**Fig. S13** HR-TEM and elemental mapping of CoFe LMH-3.


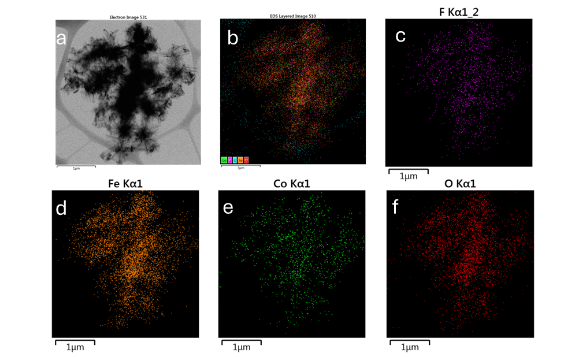


**Fig. S14** HR-TEM and elemental mapping of F-CoFe LMH-8.


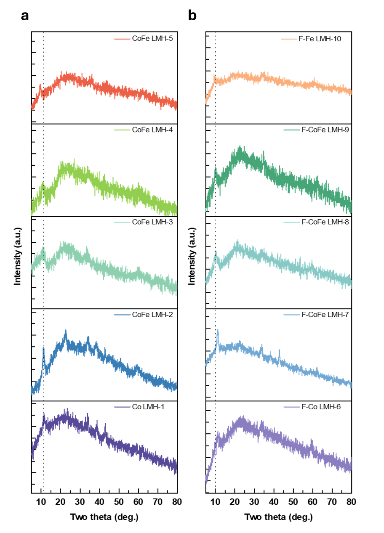


**Fig. S15** Powder-XRD analysis of as-synthesized catalysts.

**Supplementary Note S1**

XRD analysis reveals that variations in the Co and Fe concentrations in CoFe LMH lead to modifications in the diffraction profile. The (003) basal reflection exhibits a gradual shift toward lower 2θ values with changing metal composition, indicating systematic changes in the layered structure. For the CoFe LDH series, the (003) peak is observed at 11.468° for CoFe LMH-1, 11.24° for CoFe LMH-2, 10.91° for CoFe LMH-3, 9.361° for CoFe LMH-4, and 9.368° for CoFe LMH-5.

A comparable trend is observed for the fluorine-containing CoFe LDH samples (F-CoFe LMH), where the (003) reflection appears at 11.142° for F-CoFe LMH-6, 11.14° for F-CoFe LMH-7, 10.46° for F-CoFe LMH-8, 9.81° for F-CoFe LMH-9, and 9.361° for F-CoFe LMH-10. The consistent shift toward lower diffraction angles across both series suggests a composition-dependent structural changes occurring on CoFe framework.


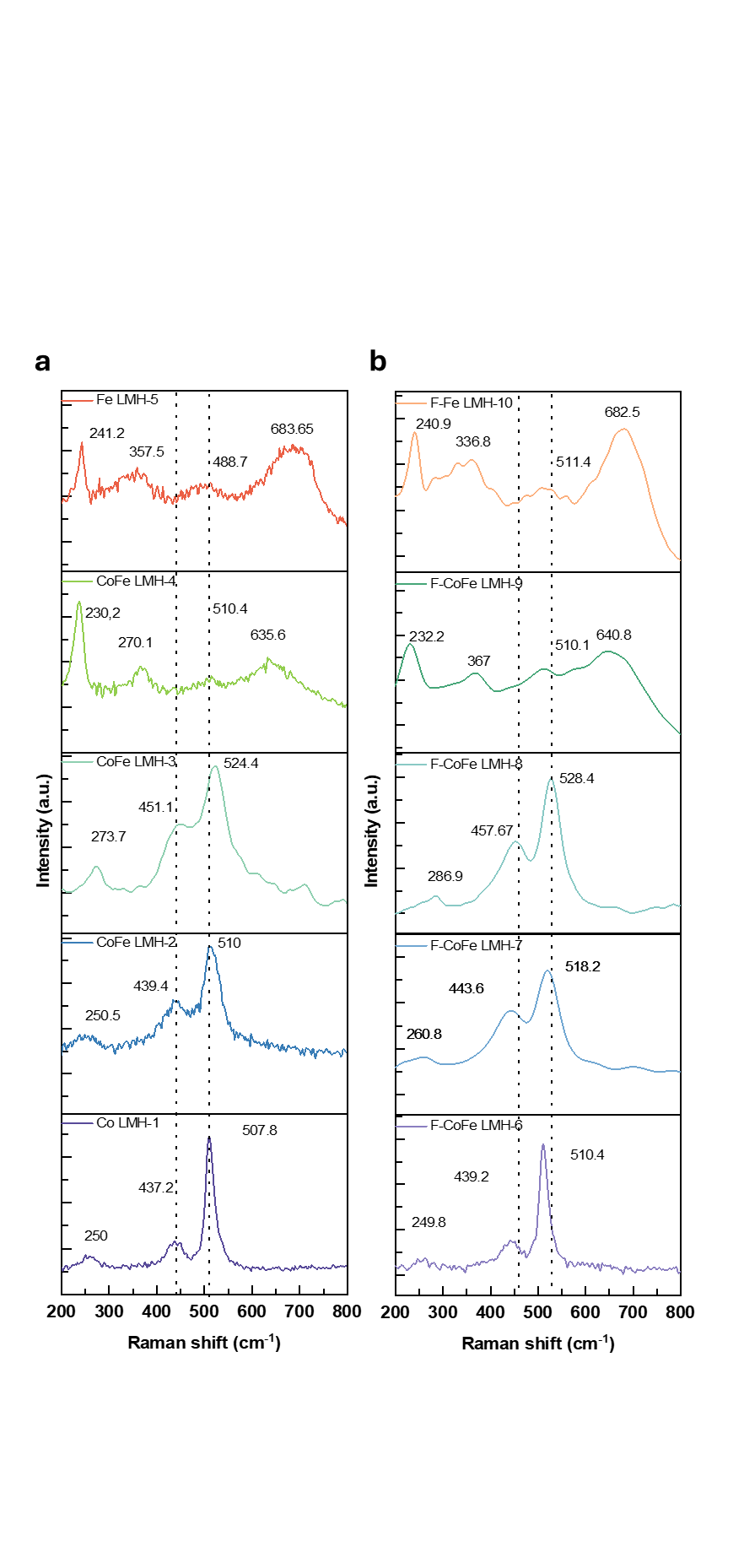


**Fig. S16** Raman analysis of as-synthesized catalysts.

**Supplementary Note S2**

The pristine catalysts exhibit distinct Raman bands corresponding to metal–oxygen lattice vibrations and hydroxyl-related modes. Co LMH-1 shows peaks at 250, 437.2, and 507.8 cm⁻¹, while CoFe LMH samples display progressive shifts with increasing Fe incorporation: LMH-2 (250.5, 439.4, 510 cm⁻¹), LMH-3 (273.7, 451.1, 524.4 cm⁻¹), and LMH-4 (230.2, 270.1, 510.4, 635.6 cm⁻¹). Fe LMH-5 exhibits broader spectral features at 241.2, 357.5, 488.7, and 683.65 cm⁻¹, indicating enhanced Fe–O vibrational contributions.

For the fluorinated series, F-CoFe LMH samples retain similar vibrational profiles with slight peak shifts, suggesting lattice distortion induced by F incorporation. F-CoFe LMH-6, LMH-7, and LMH-8 show peaks at (249.8, 439.2, 510.4), (260.8, 443.6, 518.2), and (286.9, 457.67, 528.4 cm⁻¹), respectively. F-CoFe LMH-9 and F-Fe LMH-10 exhibit additional high-frequency modes at 640.8 and 682.5 cm⁻¹, consistent with strengthened M–O–F interactions and modified hydroxyl environments.


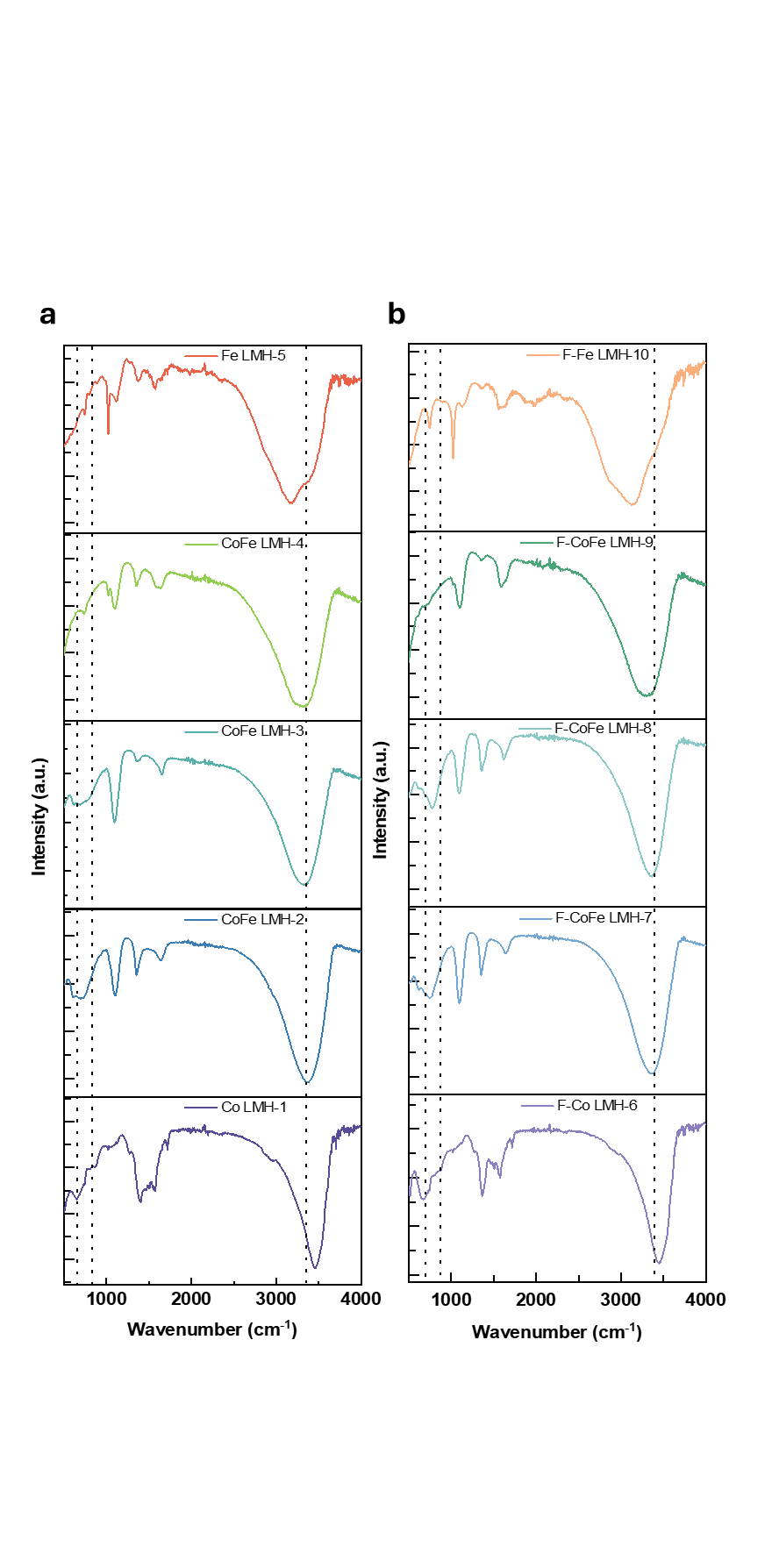


**Fig. S17:** FT-IR analysis of as-synthesized catalysts.

**Supplementary Note S3**

The FTIR spectra of the pristine series (Fig. a) and fluorinated series (Fig. b) exhibit characteristic vibrational modes associated with metal hydroxides. Co LMH-1 and CoFe LMH-2, LMH-3, LMH-4 exhibit strong, broad O-H stretching bands centered near ~3430–3450 cm⁻¹, indicating extensive hydrogen bonding among hydroxyl groups and interlayer water. Fe LMH-5 shows a slightly shifted and broadened band, suggesting stronger hydrogen bonding due to higher Fe content, the shift is consistent with F doped samples as well. Suggesting that the Fe content, alters the H bonding. A sharp feature near ~1630 cm⁻¹, attributed to H-O-H bending of adsorbed water molecules. In the low-frequency region (below 1000 cm⁻¹), distinct metal–oxygen lattice vibrations are observed. For the pristine series, Co LMH-1 shows well-defined bands, while CoFe LMH samples (LMH-2 to LMH-4) exhibit additional features and slight shifts, indicating Fe incorporation and structural distortion. Fe LMH-5 presents broader and more intense low-frequency bands, consistent with dominant Fe-O interactions. The fluorinated series (Fig. b) retains similar hydroxyl-related bands but shows subtle changes in intensity and position of metal-oxygen vibrations, suggesting lattice modification due to F doping. F-CoFe LMH samples (LMH-6 to LMH-9) exhibit sharper low-frequency peaks compared to their pristine counterparts, while F-Fe LMH-10 shows pronounced absorption features.


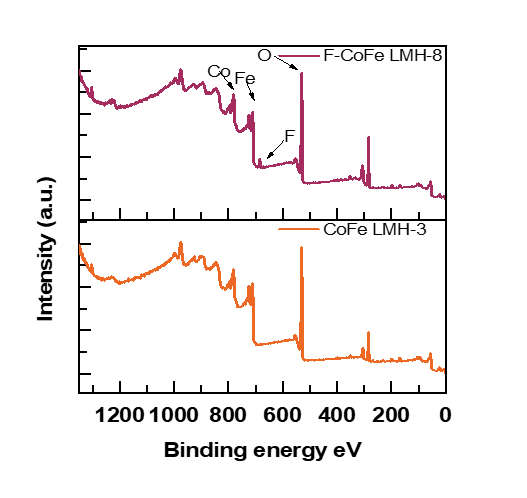


**Fig. S18** XPS survey spectrum of CoFe LMH and F-CoFe LMH.

**Fig. S19** ^19^F NMR for F-CFLMH-8.


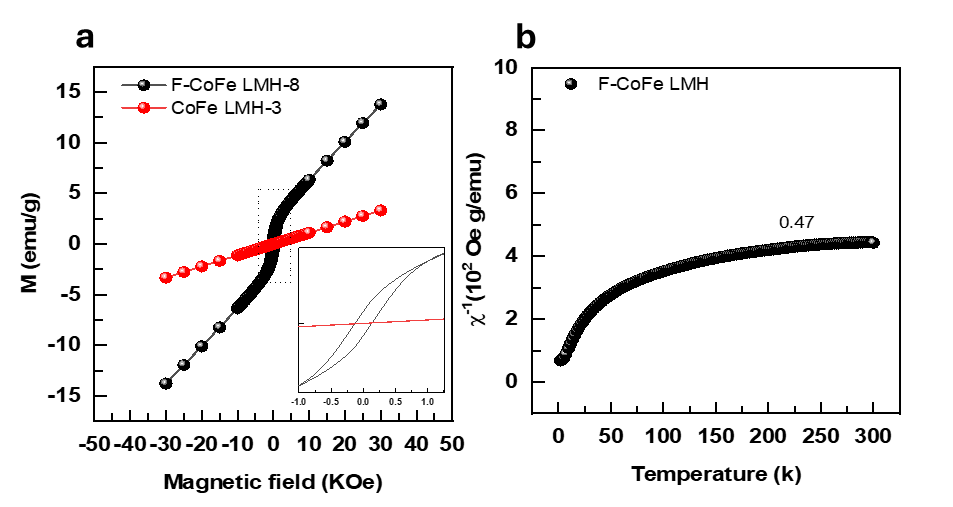


**Fig. S20** Magnetic hysteresis (M-H) loops, of (a) CoFe LMH and F-CoFe LMH. (b) Temperature-dependent susceptibility reciprocal 1/𝜒 of F-CoFe LMH.

**Supplementary Note S4**

The field-cooling (FC) temperature-dependent magnetization (M-T) characterization for powder samples using a SQUID magnetometer was carried out to further analyze the electronic configuration of the metal 3d-orbitals. in an applied field of μ_0_H = 500 Oe with a temperature range of 2-300 K. By fitting the M-T curve using the Curie-Weiss law, the effective magnetic moment (μ_eff_) can be calculated according to the equation:

$\mu_{eff}=\sqrt{\frac{3K_{B}}{N_{A}{\mu^{2}}_{B}}} \sqrt{C_{m}}$ (S17)

Where μ_eff_ is the effective magnetic moment, C_m_ is the molar curie constant k_B_ is the Boltzmann constant N_A_ is the Avogadro’s number [S14, S15].

The effective magnetic moment (μ_eff_) was found for F-CoFe LMH was found to be 4.125 μB.


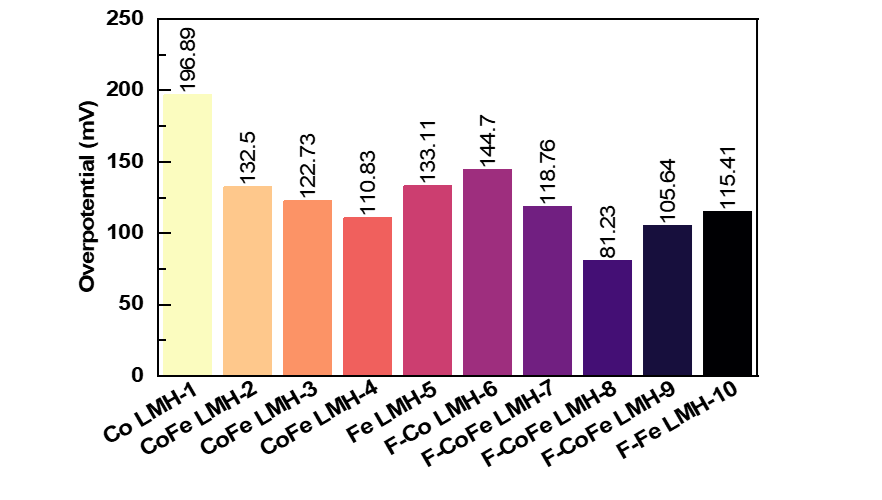


**Fig. S21** Comparison bar chart for HER overpotential.


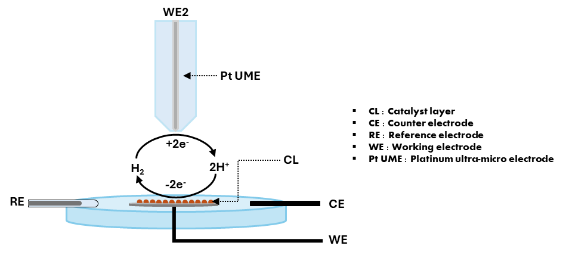


**Fig. S22** Schematic illustration of SECM over HER.


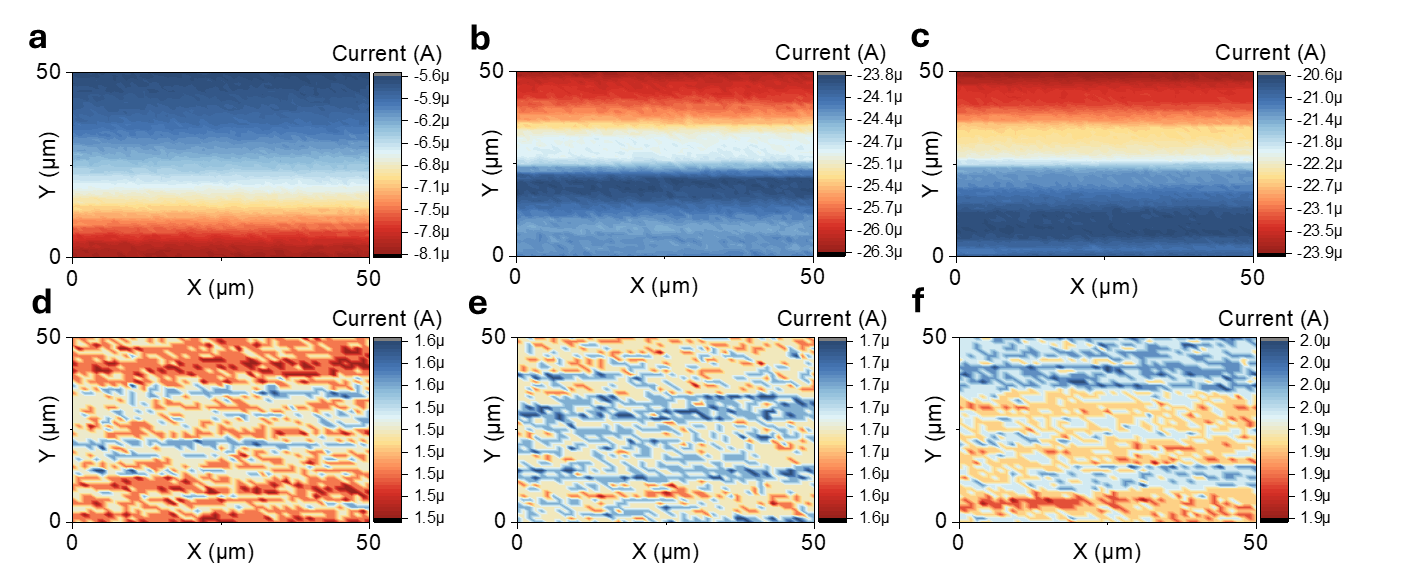


**Fig. S23** SECM analysis of CoFe LMH-3 at various current densities of -1.05 (a), -1.1 (b),-1.15 (c) vs Ag/AgCl and (d,f,g) their corresponding tip potentials.


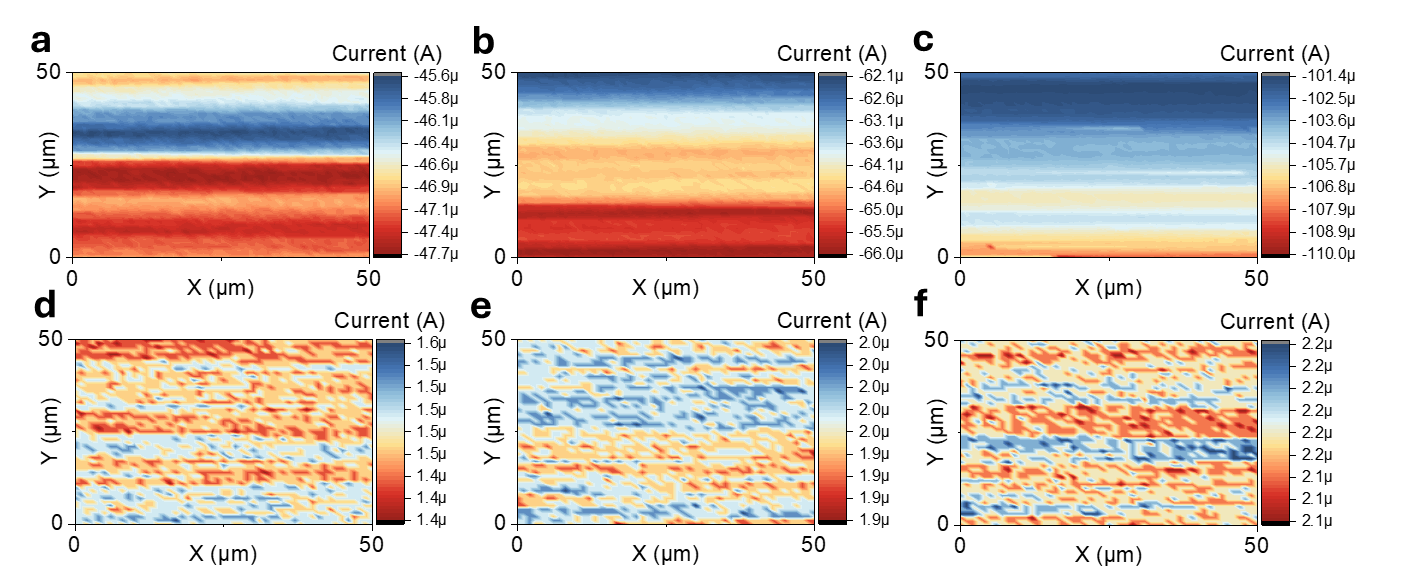


**Fig. S24** SECM analysis of F-CoFe LMH-8 at various current densities of -1.05 (a), -1.1 (b),-1.15 (c) vs Ag/AgCl and (d,f,g) their corresponding tip potentials.

**Fig. S25** HER LSV of CoFe LMH-8 before and after stability.


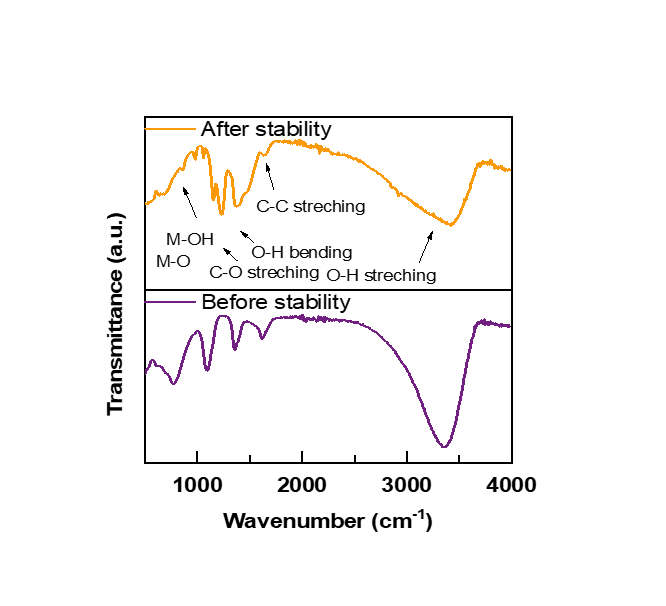


**Fig. S26** FTIR analysis of F-CoFe LMH-8 cathode before and after stability.

**Fig. S27** Raman analysis of F-CoFe LMH-8 cathode before and after stability.


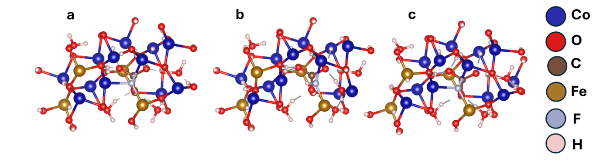


**Fig. S28** Top view of H* adsorption models for (a) Co (b) Fe (c) O.

**Fig. S29** OER overpotential and Tafel slope comparison.


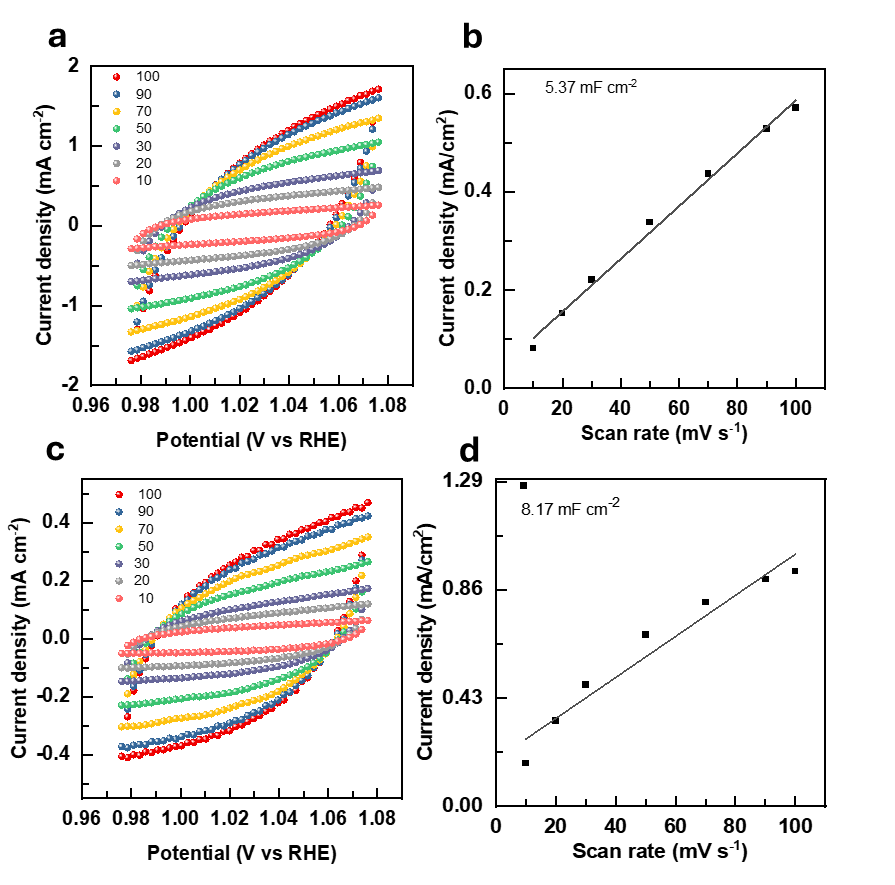


**Fig. S30** (a) CV at various scan rates for CoFe LMH-3 and (b) the corresponding electrochemical double layer capacitance. CV at various scan rates for (c) F-CoFe LMH-8 and (d) their corresponding double layer capacitance.


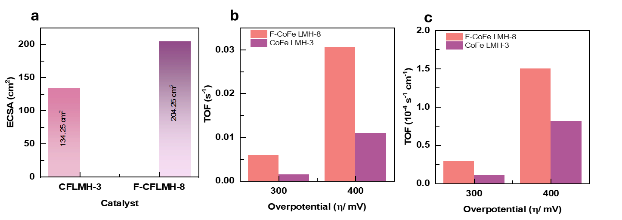


**Fig. S31** Comparative analysis of **a** ECSA analysis, **b** turnover frequency and **c** ECSA normalized turnover frequency.

**Supplementary note S5**

The turnover frequency of is used to determine the intrinsic catalyst activity of the electrocatalyst, which can be written as described by equation (S18).

$TOF=\frac{j\times A\times N_{A}}{n\times F\times M^{*}}$ (S18)

Where j is the current density (mA/cm^2^), A represents the geometric area, N_A_ is the Avogadro’s number (6.023 x 10^23^ mol^-1^), n is the number of electrons, F is the Faraday constant (96485 C mol^-1^) and M* is the total number of active sites.

The intrinsic OER performance was evaluated via ECSA and turnover frequency (TOF) analyses (Fig. S31). F-CoFe LMH-8 exhibited a larger ECSA (204.25 cm²) than pristine CoFe LMH-3 (134.25 cm²), indicating increased active site exposure. Crucially, F-CoFe LMH-8 demonstrated a significantly higher turnover frequency (TOF) at 300 mV (0.00589 s⁻¹ vs. 0.00147 s⁻¹) and 400 mV (0.03062 s⁻¹ vs. 0.01091 s⁻¹). To accurately reflect catalytic intrinsic activity, ECSA normalization was performed to TOF, yielding an ECSA-normalized TOF of 2.883 × 10⁻⁵ s⁻¹ cm⁻² for F-CoFe LMH-8 at 300 mV compared to 1.098 × 10⁻⁵ s⁻¹ cm⁻² for CoFe LMH-3 (and 1.499 × 10⁻⁴ vs. 8.126 × 10⁻⁵ s⁻¹ cm⁻² at 400 mV, respectively). These results confirm that F-doping enhances both the total number of accessible active sites and their individual intrinsic catalytic efficiency.


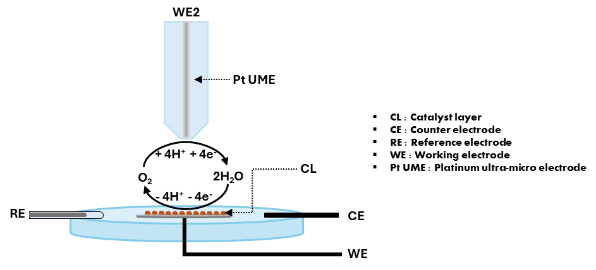


**Fig. S32** Schematic illustration of SECM over OER.


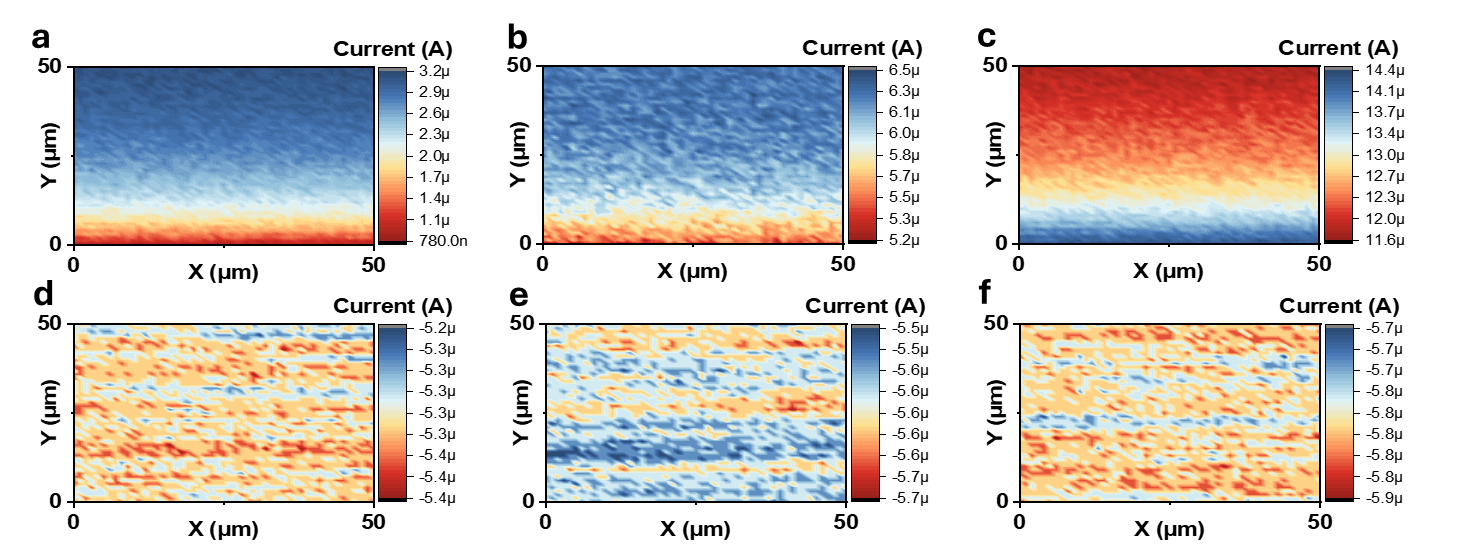


**Fig. S33** SECM analysis of CoFe LMH-3 at various current densities of 0.3V (a), 0.35V (b), and 0.4V (c) vs Ag/AgCl and (d,f,g) their corresponding tip potentials.


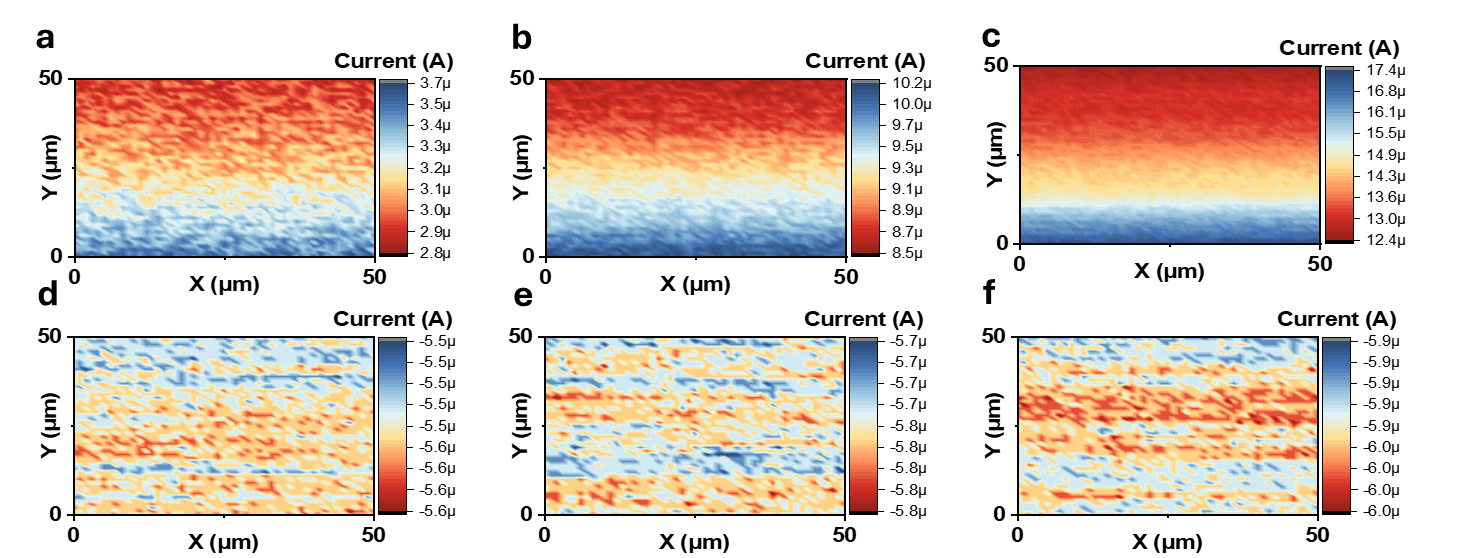


**Fig. S34** SECM analysis of F-CoFe LMH-8 at various current densities of 0.3V (a), 0.35V (b), and 0.4V (c) vs Ag/AgCl and (d,f,g) their corresponding tip potentials.

**Fig. S35** LSV for OER before and after chronopotentiometric stability analysis.


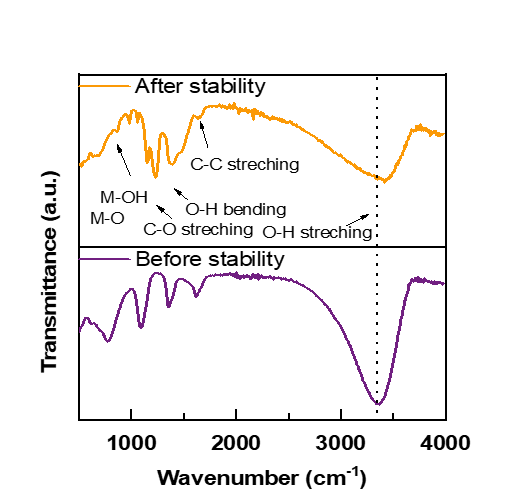


**Fig. S36** FTIR analysis of F-CoFe LMH-8 anode before and after stability.

**Fig. S37** Raman analysis of F-CoFe LMH-8 anode before and after stability.


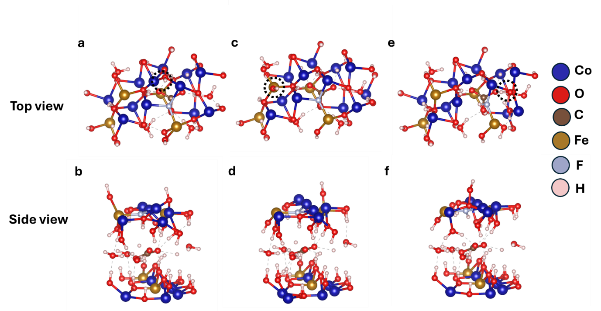


**Fig. S38** The top and side view of the optimized adsorption sites for OH* for Co, Fe and O sites respectively.


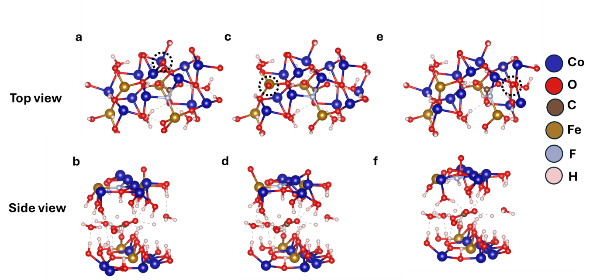


**Fig. S39** The top and side view of the optimized adsorption sites for O* for Co, Fe and O sites respectively.


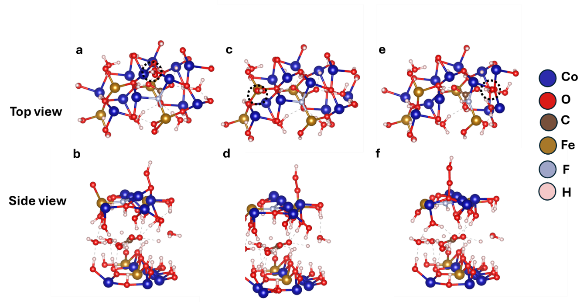


**Fig. S40** The top and side view of the optimized adsorption sites for OOH* for Co, Fe and O sites respectively.


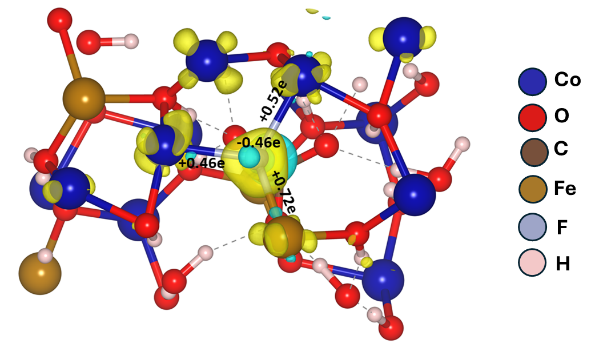


**Fig. S41** CDD of F@CoFe-LMH at ±0.005 e Å⁻³.


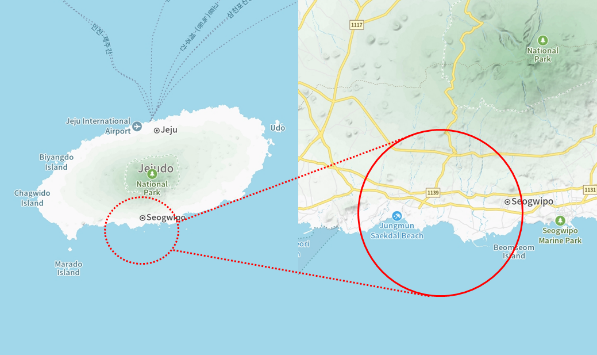


**Fig. S42** Geographical location of the seawater collected area.


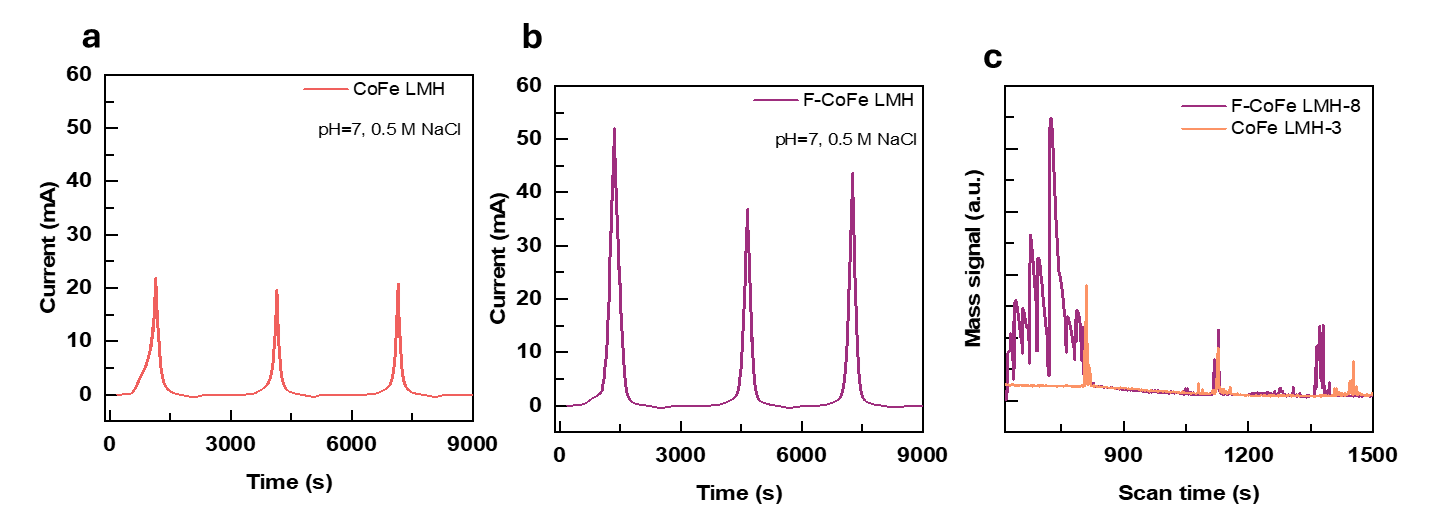


**Fig. S43** DEMS analysis of (a) CoFe LMH and (b) F-CoFe LMH and O signals in 0.5 M NaCl.


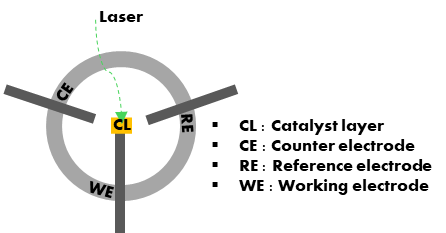


**Fig. S44** Schematic illustration for operando in-situ Raman measurements.


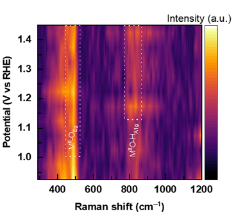


**Fig. S45** In-situ raman analysis of CoFe LMH-3.


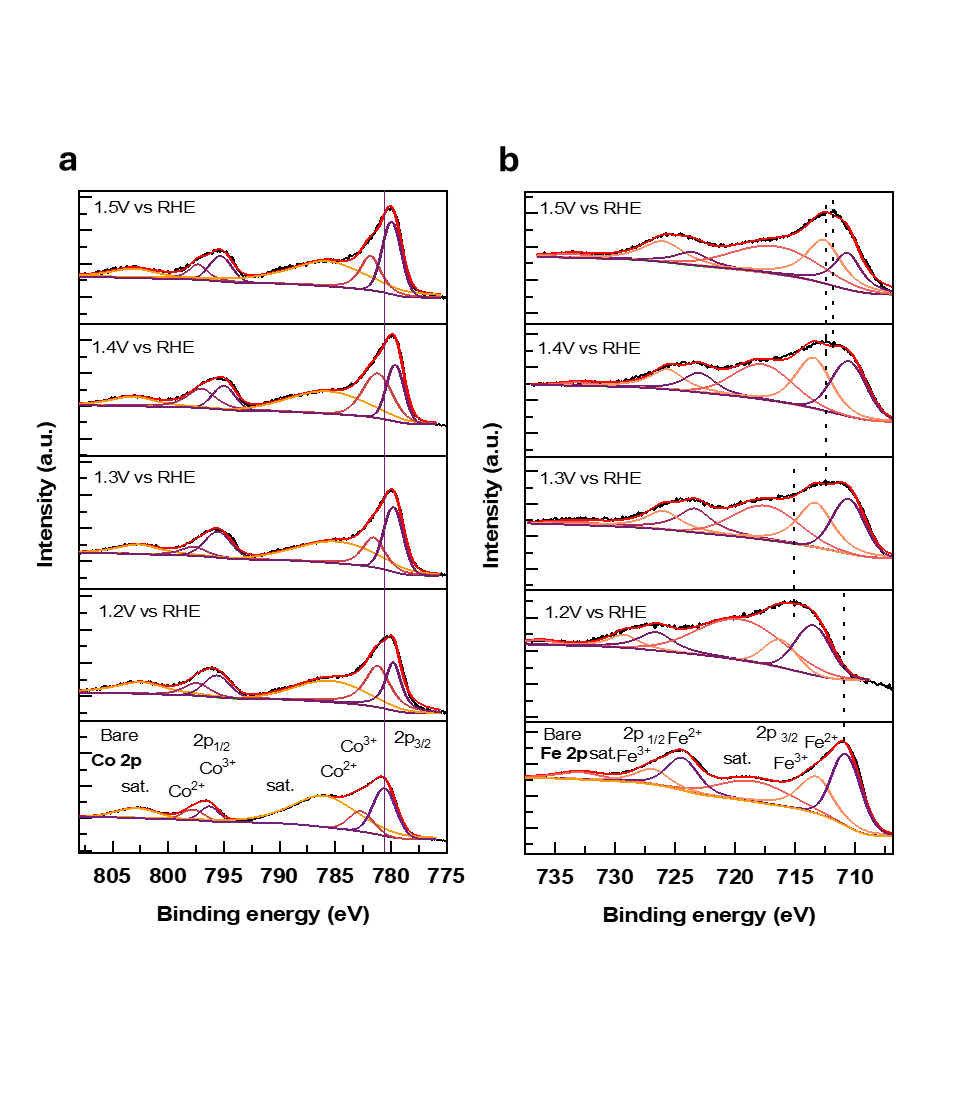


**Fig. S46** Ex-situ XPS analysis at various potentials of **a** Co 2p and **b** Fe 2p F-CoFe LMH -8.

**Supplementary note S6**

Furthermore, ex-situ XPS reveals the potential-dependent surface oxidation dynamics of the catalyst (Fig. S46). Upon anodic polarization from 1.2 to 1.5 V vs RHE, the Co 2p spectra exhibit a shift to lower energy levels, accompanied by a pronounced increase in the Co^2+^ component at 1.4 V. Beyond this potential, the Co^2+^/Co^3+^ ratio plateaus, converging toward the distribution observed at 1.2 V where Co^3+^ was initially more dominant suggesting a potential induced redox re-equilibration consistent with Co centered oxidations. In contrast, the Fe 2p region undergoes more drastic potential responsive evolution: at 1.2 V the Fe peaks shift to higher binding energy with a modest increase in Fe^3+^, whereas further polarization induces a lower energy shift that does not fully return to the initial state. This is accompanied by a substantial enrichment of Fe^3+^, as evidenced by the intensified Fe 2p_3/2_ and Fe 2p_1/2_ deconvoluted features, indicating progressive oxidation of Fe coordination environments [S16–S18]. Collectively, these correlated transitions confirm that anodic bias dynamically drives surface active sites enriched with high valence metal–oxo motifs that are requisite for O–O bond formations [S19].

**Fig. S47** Stability of F-CoFe LMH-8 in at 50 mA cm^-2^ in 1M KOH + seawater.

**
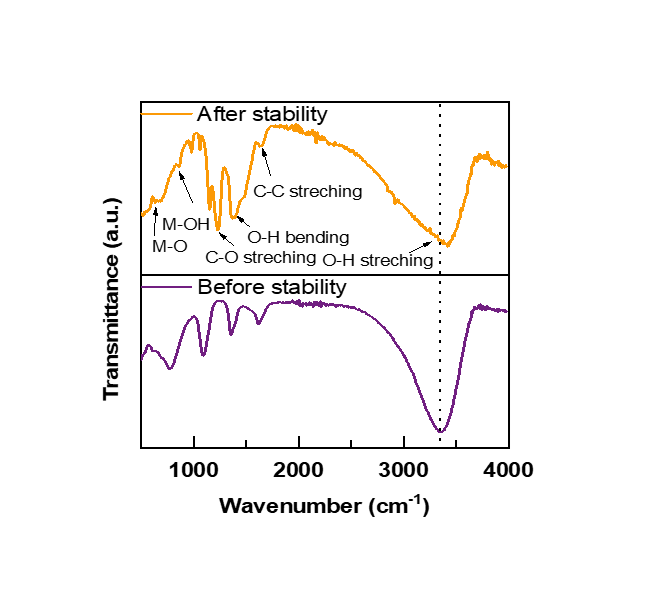
**

**Fig. S48** FTIR analysis of F-CoFe LMH-8 anode before and after stability in 1 M KOH + stimulated seawater.

**Fig. S49** XPS core level Co spectrum analysis of F-CoFe LMH-8 anode before and after stability in 1 M KOH + stimulated seawater.

**Fig. S50** XPS core level Fe spectrum analysis of F-CoFe LMH-8 anode before and after stability in 1 M KOH + stimulated seawater.

**Fig. S51** XPS core level Co spectrum analysis of F-CoFe LMH-8 anode before and after stability in 1 M KOH + stimulated seawater.


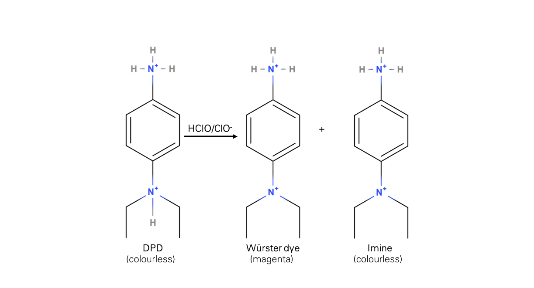


**Fig. S52** DPD analysis reaction mechanism.


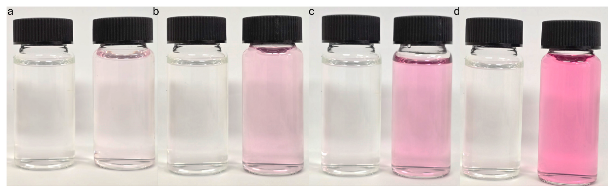


**Fig. S53** Digital images of DPD analysis of CoFe LMH-3 after stability in 1 M KOH + stimulated seawater.


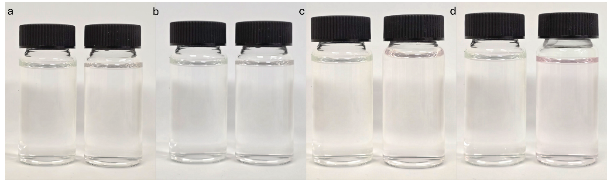


**Fig. S54** Digital images of DPD analysis of F-CoFe LMH-8 after stability in 1 M KOH + stimulated seawater.


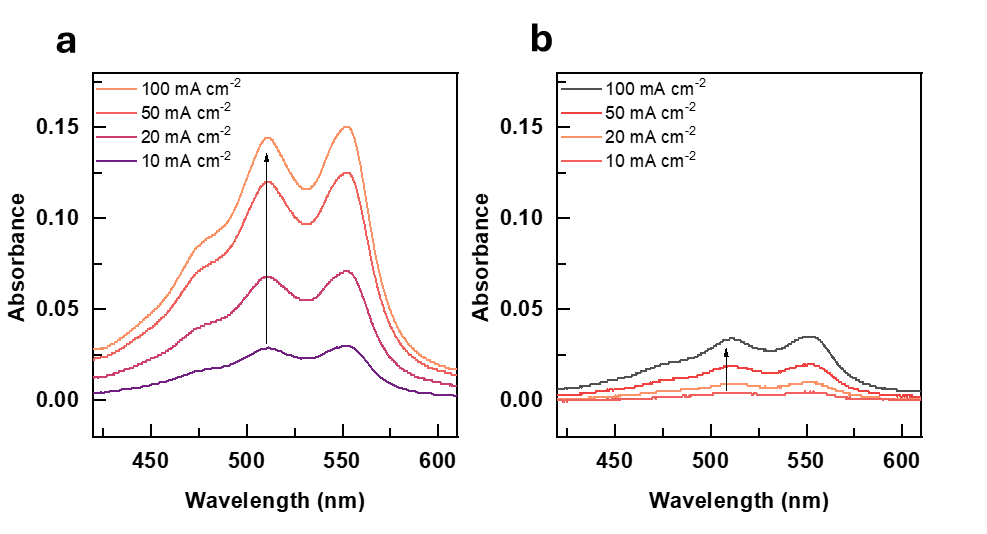


**Fig. S55** UV-Vis spectra of DPD analysis of (a) CoFe LMH-3 and (b) F-CoFe LMH-8 after analysis in 1 M KOH + stimulated seawater.

**Fig. S56** Multipotential stability of F-CoFe LMH-8 in 1M KOH + seawater.


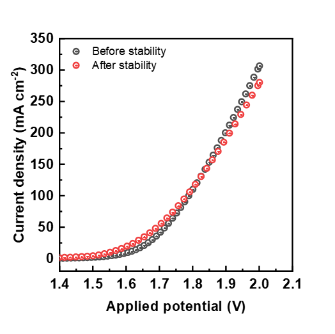


**Fig. S57** Two electrode LSV of F-CoFe LMH -8 before and after stability in multiple test scenarios (1 M + stimulated seawater → 1 M KOH + seawater).


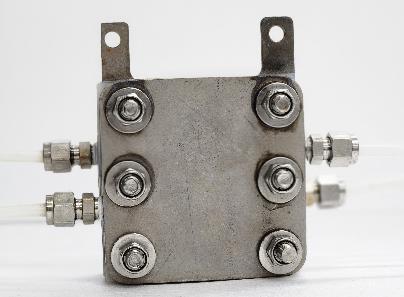


**Fig. S58** Optical photograph of electrolyzer cell used.


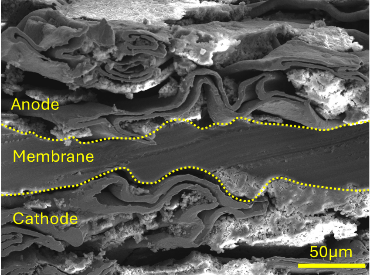


**Fig. S59** Cross-sectional FE-SEM of F-CoFe LMH -8 the prepared MEA before stability analysis.

**Fig. S60** LSV comparison of CoFe LMH-3 and F-CoFe LMH-8 in 1M KOH.


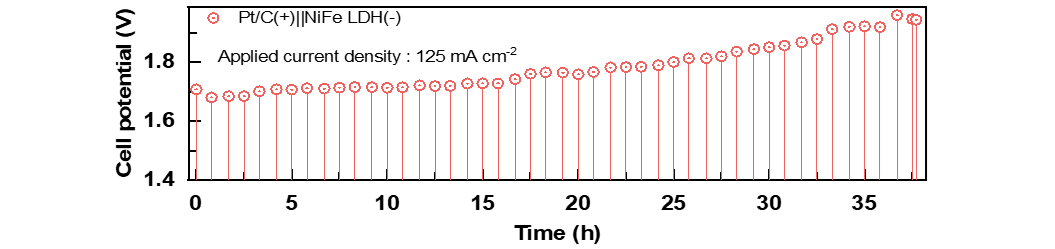


**Fig. S61** Stability of conventional NiFe LDH in 1M KOH + stimulated seawater.


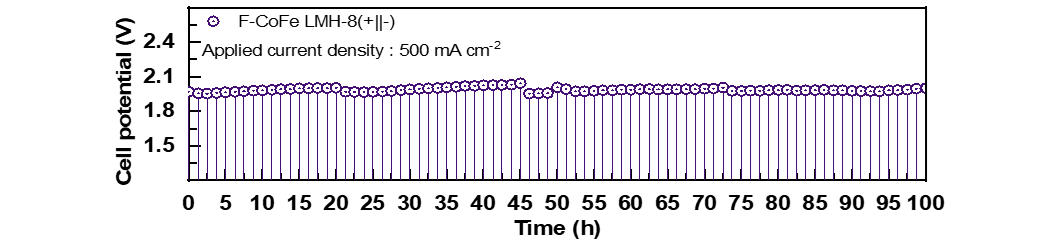


**Fig. S62** Stability of F-CoFe LMH (+/-) in 1M KOH + stimulated seawater.


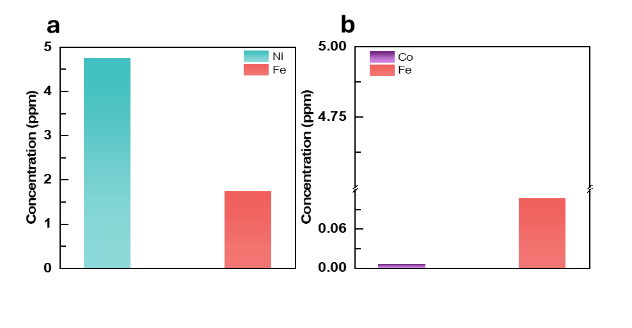


**Fig. S63** ICP-MS after stability of (a) NiFe LDH @ 125 mA/cm^2^ (b) F-CoFe-8 LDH @ 500 mA/cm^2^ analyzed in 1 M KOH + stimulated seawater.

**
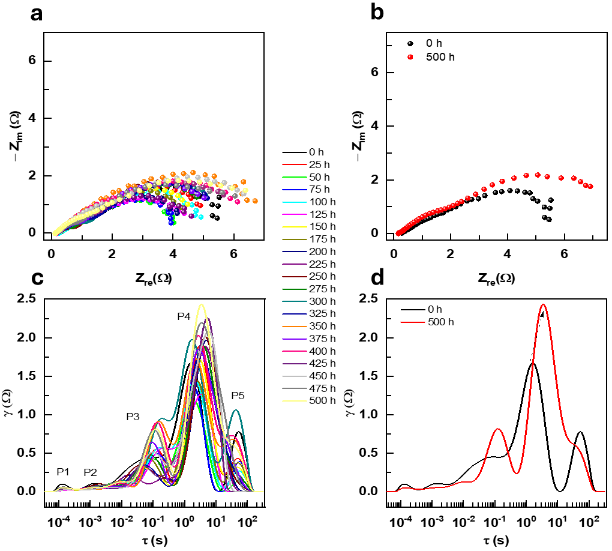
**

**Fig. S64** Time resolved (a, b) Nyquist plot and (c, d) DRT analysis during long term stability analysis in 1 M KOH + seawater.

**Supplementary note S7:**

The EIS spectra were recorded at 25 h intervals for 500 h at a fixed potential of 1.5 V analyzed from 10 kHz to 0.01 Hz at room temperature, the overall Nyquist plot is presented in Fig. S63a. After initial reconfiguring of the material, the catalyst remains stable throughout the 500 h of operational time, and only a minor increase in R_ct_ is observed thereafter, indicating its strong resistance to degradation (Fig. S63b). To further analyze the impedence behaviour DRT frequency mechanism map was outlined derived from the EIS analysis as presented in Fig. 63c. The relaxation distribution was separated into 5 distinct regions namely P1, P2, P3, P4 and P5. The regions signify P1/P2 – membrane OH^−^ conduction; P3 – cathodic charge transfer; P4 – anodic charge transfer; P5 – mass transport limitation. Improvements in OH⁻ conduction and only slight increases in the P3 and P4 regions corresponding to the cathodic and anodic stability, collectively confirming the robust and stable electrochemical performance of the F-CoFe LMH-8 MEA throughout long‑term operation” [S20].


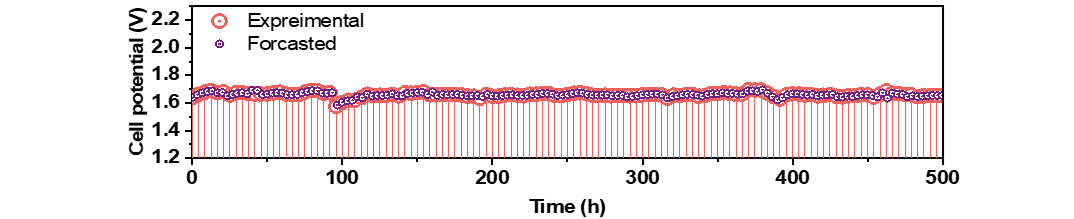


**Fig. S65** LSTM-based forecasting of the stability of F-CoFe LMH-8.

**Supplementary note S8**

The LSTM architecture can solve the vanishing gradient problem and overcome the shortcomings of conventional RNNs. Given this, it can capture long-range relationships. Memory cells and gating mechanisms are combined in LSTMs to enable selective information storing, updating, and retrieval over long periods [S15]. The LSTM modelling choice for many applications such as speech recognition, natural language processing, and time series prediction, owing to the fact that it can precisely model temporal dependencies in sequential data [S16, S17]. LSTM models are particularly useful for analyzing time-dependent data, such as voltage data of the CoFe LMH-8. The models can recognize patterns of voltage that change over time. By understanding such trends, LSTMs can help to make more accurate predictions about the CoFe LMH-8 future performance. In this case, the voltage data of CoFe LMH-8 is stationary. In other words, it doesn't fluctuate over time, so the model’s predictions focus on the average behaviour of the voltage. To improve the model’s performance, hyperparameter tuning was carried out. The *Adam* optimizer was chosen to adjust the model's weights efficiently. The model was trained over 50 epochs, with each batch containing 1000 data points. The activation functions *tanh* and *sigmoid* were used to capture the complex relationships in the data. The experimental and forecasted voltage values are shown in Fig. S65. Furthermore, the model accurately predicts the voltage of CoFe LMH-8 over the next 50 hours, as shown in Fig. 6j. The experimental and forecasted values are closely associated, and no usual deviation was observed. The model's effectiveness was measured by the mean squared error (MSE), which indicates how well the model's predictions match with experimental values. In this case, the MSE of predictions was found to be 4.69×10^−6^, which is very small. The present LSTM model is quite accurate, as a lower MSE value reflects better prediction performance. This suggests that the LSTM is an excellent machine-learning technique for predicting the stability of CoFe LMH-8.


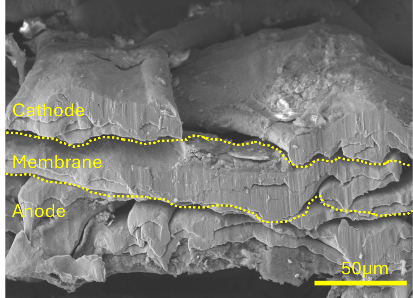


**Fig. S66** MEA of F-CoFe LMH -8 after stability in 1 M KOH + seawater.


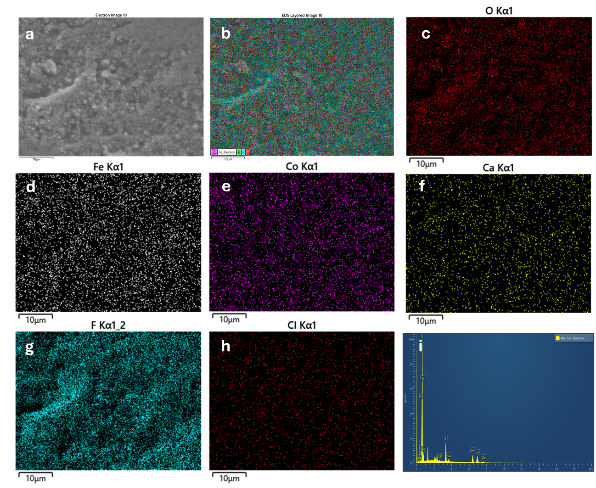


**Fig. S67** FE-SEM image, elemental mapping and EDS after stability in 1 M KOH + seawater of F-CoFe LMH-8 MEA anode.


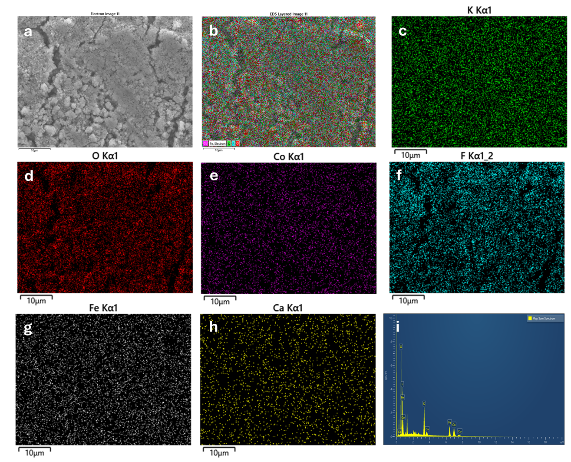


**Fig. S68** FE-SEM image, elemental mapping and EDS after stability in 1 M KOH + seawater of F-CoFe LMH-8 MEA cathode.


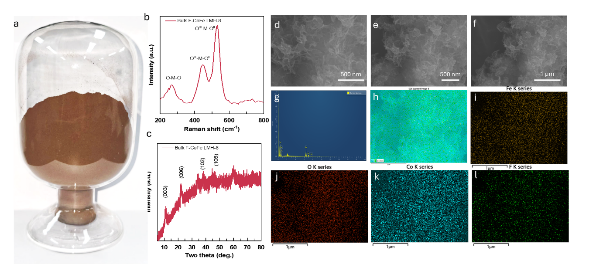


**Fig. S69** (a) Gram-scale synthesis of F-CoFe LMH, (b) Raman analysis and (c) XRD analysis and (d-f) its corresponding FE-SEM analysis, (g) EDAX and (h-l) corresponding elemental mapping.


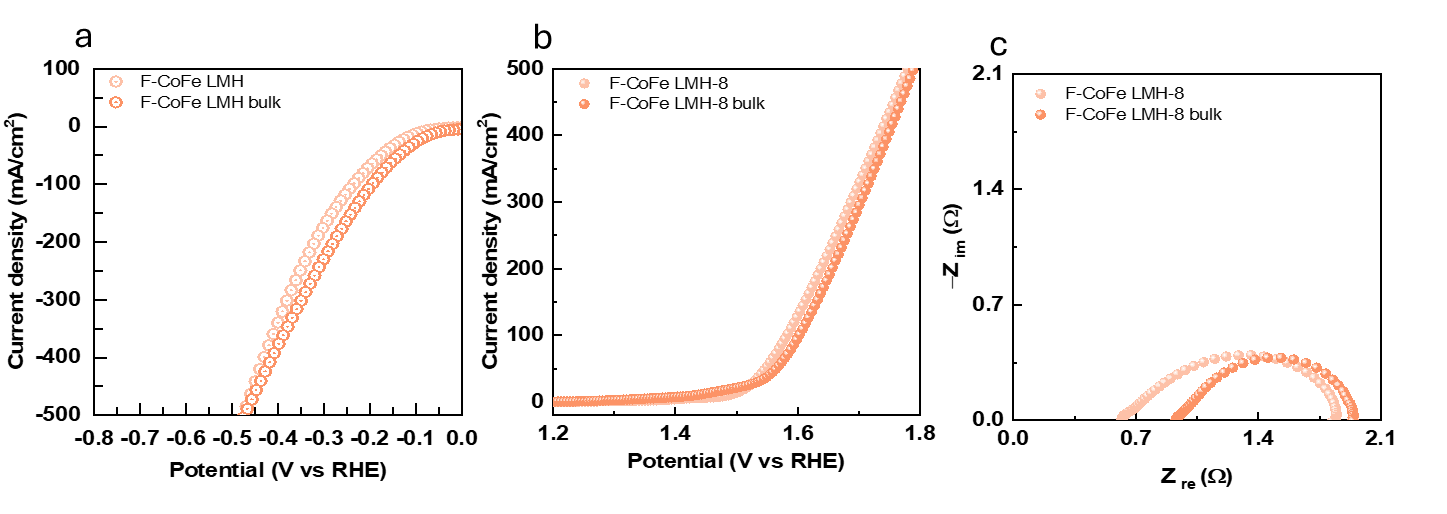


**Fig. S70** LSV comparison analysis for F-CoFe LDH-8 and F-CoFe LDH bulk in (a) 1M KOH for cathode half cell, (b) anode half cell LV and its corresponding (c) Nyquist plot in 1M KOH + 0.5 M NaCl.


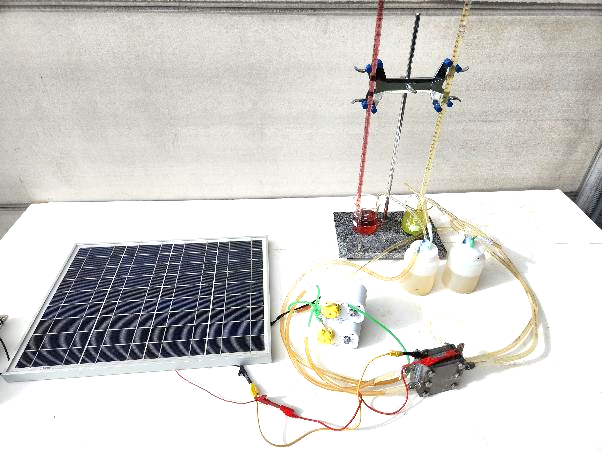


**Fig. S71** Solar cell powered seawater electrolyzer.

**Supplementary Tables**

**Table S1** Synthesis procedure for CoFe LMH

| **Catalyst name** | **CoCl_2_(mmol)** | **FeSO_4_ (mmol)** | **NH_4_F (M)** | **MgO NPs (mmol)** |
| --- | --- | --- | --- | --- |
| C-LMH-1 | 1 | 0 | - | 50 |
| CF-LMH-2 | 0.75 | 0.25 | - | 50 |
| CF-LMH-3 | 0.5 | 0.5 | - | 50 |
| CF-LMH-4 | 0.25 | 0.75 | - | 50 |
| F-LMH-5 | 0 | 1 | - | 50 |
| F-C-LMH-6 | 1 | 0 | 1 | 50 |
| F-CF-LMH-7 | 0.75 | 0.25 | 1 | 50 |
| F-CF-LMH-8 | 0.5 | 0.5 | 1 | 50 |
| F-CF-LMH-9 | 0.25 | 0.75 | 1 | 50 |
| F-F-LMH-10 | 0 | 1 | 1 | 50 |

**Table S2** HER comparison table.

| **Catalyst name** | **Overpotential(mV)** | **Current density** | **Electrolyte** | **Refs.** |
| --- | --- | --- | --- | --- |
| NiFe-LDH@CoMo–P | 49 | 10 | 1 M KOH | [S24] |
| CoFe@NiFe LDH | 256 | 100 | 1 M KOH | [S17] |
| S-NiFe-LDH | 171 | 10 | 1 M KOH | [S25] |
| CuFe-LDH/NiOOH | 101.2 | 10 | 1 M KOH | [S26] |
| CoS_2_@CoFe-LDH | 311 | 100 | 1 M KOH | [S27] |
| Ni_2_P–FeNi-LDH | 230 | 100 | 1 M KOH | [S28] |
| RuO_2_/MgFe-LDH | 122 | 10 | 1 M KOH | [S29] |
| Ru/CoOOH@NF | 139 | 10 | 1 M KOH | [S30] |
| Ag-NiCrLDH-0.3 | 116.5 | 10 | 1 M KOH | [S31] |
| NiCoB/CuFe−LDH | 129.1 | 10 | 1 M KOH | [S32] |

**Table S3** OER comparison table

| **Catalyst name** | **Overpotential(mV)** | **Current density (mA cm^-2^)** | **Electrolyte** | **Refs.** |
| --- | --- | --- | --- | --- |
| CoFe@NiFe LDH | 280 | 100 | 1 M KOH | [S17] |
| S-NiFe-LDH | 256 | 10 | 1 M KOH | [S25] |
| CuFe-LDH/NiOOH | 268.5 | 10 | 1 M KOH | [S26] |
| CoS_2_@CoFe-LDH | 450 | 100 | 1 M KOH | [S27] |
| Ni_2_P–FeNi-LDH | 270 | 100 | 1 M KOH | [S28] |
| RuO2/MgFe-LDH | 273 | 10 | 1 M KOH | [S29] |
| Ag-NiCrLDH-0.3 | 275.5 | 10 | 1 M KOH | [S31] |
| NiCoB/CuFe−LDH | 235.6 | 10 | 1 M KOH | [S32] |
| Ru/CoOOH@NF | 264 | 10 | 1 M KOH | [S30] |
| NiCo-LDH | 253 | 10 | 1 M KOH | [S33] |

**Table S4** Seawater electrolyzer comparison

| **Cathode(+)\|\|Anode(-)** | | **Performance (V @A)** | **Separator** | **Temperature (^o^C)** | **Electrolyte** | **Stability (h)** | **Ref.** |
| --- | --- | --- | --- | --- | --- | --- | --- |
| F-CoFe LMH-8\|\|F-CoFe LMH-8 | 2.14@1 | | Fumacep FAAM-15 | 50 | 1M KOH | - | ^This work^ |
| F-CoFe LMH-8\|\|F-CoFe LMH-8 | 2.3@1.2 | | Fumacep FAAM-15 | 50 | 1M KOH+ 0.5M NaCl | - | ^This work^ |
| F-CoFe LMH-8\|\|F-CoFe LMH-8 | 2.3@1.0 | | Fumacep FAAM-15 | 50 | 1M KOH+ seawater | 500 | ^This work^ |
| NiFe LDH\|\|NiFe LDH | 1.7@0.5 | | AEM | 80 | 1M KOH +seawater | 25 | [S34] |
| PW_12_-CoFe LDH | 1.99@0.5 | | PiperION-A60 | 80 | 1M KOH +seawater | 1000 | [S35] |
| Raney NiPO_4_^3–^/NiFe-LDH | 2.0@1 | | Diaphram | 60 | 1 M KOH + seawater | 1000 | [S36] |
| SO_4_^2−^/CoFe LDH | 2.264@0.5 | | - | RT | 1 M KOH + seawater | 150 | [S37] |
| Raney Ni \|\| NiFe-LDH/NiMoO_4_ | 1.847@1 | | UTP 220 diaphram | 80 | 1 M KOH + seawater | 50 | [S38] |
| FeCoP/TiN/CP\|\|NiFe-LDH | 1.79@0.5 | | AEM | 60 | 1 M KOH + seawater | 100 | [S39] |
| Pt\|\|F-NiFe LDH | 2.04@0.5 | | AEM | RT | 1 M KOH + seawater | 80 | [S40] |
| Pt/C\|\| MoO_3_@CoO | 1.99@2 | | Amphoteric membranes | 60 | 6 M KOH + seawater | 500 | [S41] |
| NiSx\|\| Cl-NiFe LDH | 1.9@0.4 | | - | RT | 1 M KOH + seawater | 100 | [S42] |
| Pt/C\|\| SS-NiFe-60 | 2.25@0.1 | | sustanion | RT | 1.0 M KOH + 0.6 M NaCl | 100 | [S43] |

**Table S5** Apparent concentration of various ions extracted from EDS analysis of F-CoFe LMH-8 MEA cathode

| **Element** | **Apparent Concentration** |
| --- | --- |
| **Ca** | 1.92 |
| **Fe** | 26.09 |
| **Co** | 26.54 |

**Table S6** Apparent concentration of various ions extracted from EDS analysis of F-CoFe LMH-8 MEA anode

| **Element** | **Apparent Concentration** |
| --- | --- |
| **O** | 47.09 |
| **Cl** | 0.44 |
| **Fe** | 19.53 |
| **Co** | 21.22 |

**Supplementary References**

1. M. Saccoccio, T.H. Wan, C. Chen, F. Ciucci, Optimal regularization in distribution of relaxation times applied to electrochemical impedance spectroscopy: ridge and lasso regression methods - a theoretical and experimental study. Electrochim. Acta **147**, 470–482 (2014). <https://doi.org/10.1016/j.electacta.2014.09.058>
2. A. Maradesa, B. Py, T.H. Wan, M.B. Effat, F. Ciucci, Selecting the regularization parameter in the distribution of relaxation times. J. Electrochem. Soc. **170**(3), 030502 (2023). <https://doi.org/10.1149/1945-7111/acbca4>
3. T.H. Wan, M. Saccoccio, C. Chen, F. Ciucci, Influence of the discretization methods on the distribution of relaxation times deconvolution: implementing radial basis functions with DRTtools. Electrochim. Acta **184**, 483–499 (2015). <https://doi.org/10.1016/j.electacta.2015.09.097>
4. A.S. Botana, M.R. Norman, Electronic structure and magnetism of transition metal dihalides: Bulk to monolayer. Phys. Rev. Mater. **3**(4), 044001 (2019). <https://doi.org/10.1103/physrevmaterials.3.044001>
5. G. Kresse, J. Hafner, Norm-conserving and ultrasoft pseudopotentials for first-row and transition elements. J. Phys.: Condens. Matter **6**(40), 8245–8257 (1994). <https://doi.org/10.1088/0953-8984/6/40/015>
6. S. Ehrlich, J. Moellmann, W. Reckien, T. Bredow, S. Grimme, System-dependent dispersion coefficients for the DFT-D3 treatment of adsorption processes on ionic surfaces. Chemphyschem **12**(17), 3414–3420 (2011). <https://doi.org/10.1002/cphc.201100521>
7. P.E. Blöchl, O. Jepsen, O.K. Andersen, Improved tetrahedron method for Brillouin-zone integrations. Phys. Rev. B **49**(23), 16223–16233 (1994). <https://doi.org/10.1103/physrevb.49.16223>
8. V. Wang, N. Xu, J.-C. Liu, G. Tang, W.-T. Geng, VASPKIT: a user-friendly interface facilitating high-throughput computing and analysis using VASP code. Comput. Phys. Commun. **267**, 108033 (2021). <https://doi.org/10.1016/j.cpc.2021.108033>
9. B. Hammer, J.K. Nørskov, Electronic factors determining the reactivity of metal surfaces. Surf. Sci. **343**(3), 211–220 (1995). <https://doi.org/10.1016/0039-6028(96)80007-0>
10. D. Zhou, Z. Cai, Y. Jia, X. Xiong, Q. Xie et al., Activating basal plane in NiFe layered double hydroxide by Mn^2+^ doping for efficient and durable oxygen evolution reaction. Nanoscale Horiz. **3**(5), 532–537 (2018). <https://doi.org/10.1039/C8NH00121A>
11. E. Skúlason, V. Tripkovic, M.E. Björketun, S. Gudmundsdóttir, G. Karlberg et al., Modeling the electrochemical hydrogen oxidation and evolution reactions on the basis of density functional theory calculations. J. Phys. Chem. C **114**(42), 18182–18197 (2010). <https://doi.org/10.1021/jp1048887>
12. Z. Fu, C. Ling, J. Wang, A Ti_3_C_2_O_2_ supported single atom, trifunctional catalyst for electrochemical reactions. J. Mater. Chem. A **8**(16), 7801–7807 (2020). <https://doi.org/10.1039/d0ta01047b>
13. S. Prabhakaran, D.H. Kim, Enhanced electrocatalytic activity with an incorporation of oxygen on the surface of di-nickel di-selenide for water splitting: a DFT-based computational design. Appl. Surf. Sci. **614**, 156255 (2023). <https://doi.org/10.1016/j.apsusc.2022.156255>
14. C.E. Housecroft, A.G. Sharpe, Inorganic chemistry, 4th edn,( Pearson Education Ltd, 2012).
15. C. Kittel, Introduction to Solid State Physics, 8th edn, (John Wiley & Sons, Berkeley, New York, 1996).
16. L. Zhang, C. Han, Z. Ye, D. Cui, J. Yang et al., Mo-doped CoFe-layered double hydroxides as an efficient bifunctional electrocatalyst for overall water splitting: Effects of different molybdenum additions on catalytic performance. Int. J. Hydrog. Energy **93**, 355–363 (2024). <https://doi.org/10.1016/j.ijhydene.2024.10.420>
17. Y. Zhong, Z. Zhang, X. Chen, Q. Chen, L. Chen et al., Interface synergy engineering in CoFe@NiFe LDH hierarchical heterostructures for bifunctional water splitting. J. Alloys Compd. **1041**, 183765 (2025). <https://doi.org/10.1016/j.jallcom.2025.183765>
18. M. Rong, H. Zhong, S. Wang, X. Ma, Z. Cao, La/Ce doped CoFe layered double hydroxides (LDH) highly enhanced oxygen evolution performance of water splitting. Colloids Surf. A Physicochem. Eng. Aspects **625**, 126896 (2021). <https://doi.org/10.1016/j.colsurfa.2021.126896>
19. Q. Yang, Y. Li, A. Kong, Y. Li, T. An et al., F-Doping-driven spin reconfiguration in NiFe LDH: Unlocking lattice oxygen for high-efficiency oxygen evolution reaction. Appl. Catal. B Environ. Energy **383**, 126130 (2026). <https://doi.org/10.1016/j.apcatb.2025.126130>
20. M. Ranz, B. Grabner, B. Schweighofer, H. Wegleiter, A. Trattner, Dynamics of anion exchange membrane electrolysis: Unravelling loss mechanisms with electrochemical impedance spectroscopy, reference electrodes and distribution of relaxation times. J. Power Sources **605**, 234455 (2024). <https://doi.org/10.1016/j.jpowsour.2024.234455>
21. Y. Yu, X. Si, C. Hu, J. Zhang, A review of recurrent neural networks: LSTM cells and network architectures. Neural Comput. **31**(7), 1235–1270 (2019). https://doi.org/10.1162/neco_a_01199
22. P.A. Koyale, S.V. Mulik, J.L. Gunjakar, T.D. Dongale, V.B. Koli et al., Synergistic enhancement of water-splitting performance using MOF-derived ceria-modified g-C_3_N_4_ nanocomposites: synthesis, performance evaluation, and stability prediction with machine learning. Langmuir **40**(26), 13657–13668 (2024). <https://doi.org/10.1021/ACS.LANGMUIR.4C01336>
23. K. Sharma, K. Bhunia, S. Chatterjee, M. Perumalsamy, A.A. Saj et al., Deep learning-assisted organogel pressure sensor for alphabet recognition and bio-mechanical motion monitoring. Nanomicro Lett. **18**(1), 63 (2025). https://doi.org/10.1007/s40820-025-01912-z
24. D. Guo, H. Xia, X. Guo, L. Wen, T. Wang et al., Synthesis bifunctional catalysts with amorphous NiFe-LDH/crystalline CoMo bimetallic phosphide heterojunction by electrodeposition for efficient water splitting. Int. J. Hydrog. Energy **79**, 73–85 (2024). <https://doi.org/10.1016/j.ijhydene.2024.06.426>
25. S. Zhang, Y. Ji, S. Wang, P. Zhang, D. Shi et al., Sulfur doping induces internal polarization field in NiFe-LDH for bifunctioanl HER/OER and overall water/simulated seawater splitting. J. Alloys Compd. **1002**, 175323 (2024). <https://doi.org/10.1016/j.jallcom.2024.175323>
26. N. Ambikeswari, A. Anto Jeffery, G. Sandoval-Hevia, K. Shanmugaraj, N. Chidhambaram et al., Synergetic nanoarchitectonics with CuFe-LDH@NiOOH interface for high-efficiency bifunctional electrocatalyst toward accelerated electrocatalytic water splitting. Int. J. Hydrog. Energy **167**, 150994 (2025). <https://doi.org/10.1016/j.ijhydene.2025.150994>
27. G. Afshan, S. Karim, Y.P. Kharwar, T. Aziz, S. Saha et al., Green H_2_ generation from seawater deploying a bifunctional hetero-interfaced CoS_2_-CoFe-layered double hydroxide in an electrolyzer. Small **21**(7), 2406431 (2025). <https://doi.org/10.1002/smll.202406431>
28. Z.-Q. Ge, J. Li, H.-J. Zhang, C. Liu, G. Che et al., P–d orbitals coupling heterosites of Ni2P/NiFe-LDH interface enable O─H cleavage for water splitting (adv. funct. mater. 40/2024). Adv. Funct. Mater. **34**(40), 2470234 (2024). <https://doi.org/10.1002/adfm.202470234>
29. S. Nagappan, R. Jayan, N. Rajagopal, A.V. Krishnan, M.M. Islam et al., Tailoring Mott−Schottky RuO_2_/MgFe-LDH heterojunctions in electrospun microfibers: a bifunctional electrocatalyst for water electrolysis. Small **20**(43), 2403908 (2024). <https://doi.org/10.1002/smll.202403908>
30. Y. Ma, Y. Ha, L. Chen, Z. An, L. Xing et al., Electrochemically induced Ru/CoOOH synergistic catalyst as bifunctional electrode materials for alkaline overall water splitting. Small **20**(27), 2311884 (2024). <https://doi.org/10.1002/smll.202311884>
31. H. Ren, S. Xu, K. Jia, H. Zhou, R.-D. Zhao et al., Enhancing water/seawater electrolysis catalysis through Ag doping: High-performance and stable NiCr-LDH for bifunctional electrocatalyst. Surf. Interfaces **78**, 108075 (2025). <https://doi.org/10.1016/j.surfin.2025.108075>
32. T.P. Raj, P. Nitesh, C. Sengottaiyan, B. Neppolian, A. Seetharaman et al., Synergistic insights into the electrocatalytic mechanisms of interface engineered NiCoB@CuFe−LDH heterojunctions for enhanced water splitting. Int. J. Hydrog. Energy **173**, 151370 (2025). <https://doi.org/10.1016/j.ijhydene.2025.151370>
33. S.A. Abdelfattah, M.M. Omran, A. Mohamed, A.M. Matloob, O.E. Mahmoud, *In-situ* preparation of NiCo-LDH/NF electrode for green hydrogen production through alkaline water electrolysis. Mater. Chem. Phys. **332**, 130097 (2025). <https://doi.org/10.1016/j.matchemphys.2024.130097>
34. X. Li, S.-L. Xu, J. Li, S.-S. Zhang, B.-Y. Zhang et al., NiFe-LDH as a bifunctional electrocatalyst for efficient water and seawater electrolysis: enhanced oxygen evolution and hydrogen evolution reactions. Nanoscale Adv. **7**(18), 5546–5560 (2025). <https://doi.org/10.1039/D5NA00350D>
35. X. He, Y. Yao, M. Zhang, Y. Zhou, L. Zhang et al., Engineered PW(12)-polyoxometalate docked Fe sites on CoFe hydroxide anode for durable seawater electrolysis. Nat. Commun. **16**(1), 5541 (2025). <https://doi.org/10.1038/s41467-025-60620-9>
36. X. Sun, W. Shen, H. Liu, P. Xi, M. Jaroniec et al., Corrosion-resistant NiFe anode towards kilowatt-scale alkaline seawater electrolysis. Nat. Commun. **15**(1), 10351 (2024). <https://doi.org/10.1038/s41467-024-54754-5>
37. Y. Yu, W. Zhou, X. Zhou, J. Yuan, X. Zhang et al., Taking advantage of activation potential coincidence to unlock stable direct seawater splitting. Adv. Funct. Mater. **35**(20), 2419871 (2025). <https://doi.org/10.1002/adfm.202419871>
38. L. Wang, K. Huang, X. Zheng, Y. Liu, J. Wu et al., A corrosion-resistant amorphous/crystalline heterostructured catalyst for industrial-level seawater electrolysis in membrane electrode assembly electrolyzer. Adv. Funct. Mater. **35**(42), 2417603 (2025). <https://doi.org/10.1002/adfm.202417603>
39. X. Yang, W. Guo, H. Xi, H. Pang, Y. Ma et al., Engineering N─TM(co/Fe)─P interfacial electron bridge in transition metal phosphide/nitride heterostructure nanoarray for highly active and durable hydrogen evolution in large-current seawater electrolysis. Adv. Funct. Mater. 2505078 (2025). <https://doi.org/10.1002/adfm.202505078>
40. J. Mu, C. Yu, X. Song, L. Chen, J. Zhao et al., A super-chlorophobic yet weak-reconstructed electrocatalyst by fluorination engineering toward chlorine oxidation-free and high-stability seawater electrolysis. Adv. Funct. Mater. **35**(23), 2423965 (2025). <https://doi.org/10.1002/adfm.202423965>
41. L. Zhou, D. Guo, L. Wu, Z. Guan, C. Zou et al., A restricted dynamic surface self-reconstruction toward high-performance of direct seawater oxidation. Nat. Commun. **15**(1), 2481 (2024). <https://doi.org/10.1038/s41467-024-46708-8>
42. H. Liu, W. Shen, H. Jin, J. Xu, P. Xi et al., High-performance alkaline seawater electrolysis with anomalous chloride promoted oxygen evolution reaction. Angew. Chem. Int. Ed. **62**(46), e202311674 (2023). <https://doi.org/10.1002/anie.202311674>
43. X. Wan, J. Li, L. Lei, J. Wang, L. Zhuang et al., Chloride-resistant seawater electrolysis *via* hydroxyl network-tailored NiFe hydroxide catalysts on stainless steel. AlChE. J. **71**(8), e18872 (2025). <https://doi.org/10.1002/aic.18872>
